# The Decomposition of Between and Within Effects in Contextual Models

**DOI:** 10.3389/fpsyg.2021.541803

**Published:** 2021-06-03

**Authors:** Siwen Guo, Richard T. Houang, William H. Schmidt

**Affiliations:** ^1^Department of Psychology, Renmin University of China, Beijing, China; ^2^Center for the Study of Curriculum Policy, Department of Counseling, Educational Psychology & Special Education, Michigan State University, East Lansing, MI, United States

**Keywords:** contextual model, multilevel modeling, structural equation modeling, multilevel SEM, mediation, finite population correction

## Abstract

In contextual studies, group compositions are often extracted from individual data in the sample, in order to estimate the group compositional effects [e.g., school socioeconomic status (SES) effect] controlling for interindividual differences in multilevel models. As the same variable is used at both group level and individual level, an appropriate decomposition of between and within effects is a key to providing a clearer picture of these organizational and individual processes. The current study developed a new approach with within-group finite population correction (fpc). Its performances were compared with the manifest and latent aggregation approaches in the decomposition of between and within effects. Under a moderate within-group sampling ratio, the between effect estimates from the new approach had a lesser degree of bias and higher observed coverage rates compared with those from the manifest and latent aggregation approaches. A real data application was also used to illustrate the three analysis approaches.

## Introduction

In contextual models, the group compositional effects on individual development or outcomes and their underlying organizational processes have attracted a large amount of attention (Mayer et al., 2014). The individual-level constructs and their aggregated group compositions often show different effects on individual outcomes, which reflect different theoretical meanings (Lau and Nie, 2008; Marsh et al., 2012). The big-fish-little-pond effect is an example, which found that student academic self-concept was positively associated with individual achievement but negatively associated with school average achievement. The school-level effect of achievement on student academic self-concept reflected the way schools were structured and their effects on individuals (Marsh et al., 2009). Group compositional effects, or the effects of aggregated individual characteristics, like socioeconomic status (SES), gender, and ethnicity, etc., have drawn attention in contextual studies. The study on student and school SES effects is one good example, which examines the between-group effect of group compositions and the within-group effect of individual characteristics (Raudenbush and Bryk, 2002; Lüdtke et al., 2008). To explore the between-group and within-group effects, a two-level random intercept model (referred to as MLM model for simplicity) is often used.

The models that include the same variable at both individual- and group levels are called contextual models or compositional models (Lüdtke et al., 2008). In these models, the central question is whether the aggregated group compositions have any effect on individual outcomes controlling for interindividual differences (Marsh et al., 2009). If individuals are randomly selected from the entire population with error-free measurements of their characteristics as well as their group compositions, a single level model would work to separate and describe the effects of group compositions and individual characteristics.

Challenges arise, however, in a two-stage cluster sampling design, which is often used in data collection in these contextual studies, as individuals are naturally nested in groups. Meanwhile, the group compositions are usually unknown and need to be extracted from individual data in the sample, which generally brings sampling errors into the aggregated group compositions. As the same variable is used at both group level and individual level, an appropriate decomposition of the between-group and within-group effects^1^ is a key to providing a clearer picture of these organizational and individual processes (Zhang et al., 2009). The current study aims at assessing the performances of different analysis approaches in the decomposition of between and within effects in contextual models.

The previous contextual studies have investigated not only the between effects of group compositions and the within effects of individual characteristics on individual outcomes but also their roles as mediators as well as their indirect effects through other variables. If it was the nature of the mediating effects that occurred not only on the individual level but also on the group level, the within and between indirect effects should be identified in multilevel models. For a 2-1-1 mediation model (i.e., the treatment is measured at group level, and the mediator and outcome are measured at individual level, and referred to as 2-1-1 mediation model in the current study). In a cluster randomized design, Keenan and Laura (2012) showed that the power to detect the mediation effects was reduced when the mediation was unnecessarily restricted to the group level, and recommended testing the cross-level indirect effects in empirical studies. Tofighi and Thoemmes (2014) discussed how to specify, estimate, and interpret the results of single-level and multilevel mediation analyses for different research questions in the 2-1-1 settings. Talloen et al. (2016) further discussed the assumptions under which the within and between indirect effects could be identified and proposed a sensitivity analysis to assess the potential impact of unmeasured confounders on the within and between indirect effects.

A study on the school-based tobacco prevention programs, which aimed at lowering youth initiation of smoking via norms, is an empirical example, exploring the mediating effect of a group-level aggregated construct on the relationship between group-level treatment and individual outcomes (Pituch et al., 2006). The group norm is aggregated from individual norms, and its mediating effect is the research focus. This mediating effect is usually modeled in a 2-1-1 mediation model. Schmidt et al. (2015) examined the between and within indirect effects of SES on student mathematics achievement through the opportunity to learn (OTL). This example explores the indirect effects of aggregated group compositions and individual characteristics. The between and within indirect effects are usually modeled in a 1-1-1 mediation model (i.e., the predictor, mediator, and outcome are measured at an individual level, referred to as the 1-1-1 mediation model in the current study).

Following the research trends adopted in other contextual studies, this study discusses the decomposition of between and within effects for the MLM, 2-1-1 mediation and 1-1-1 mediation models.

## Manifest Aggregation Approach

Two modeling approaches, the manifest and latent aggregation approaches, were proposed in previous studies to decompose the between and within effects. Traditionally, the between and within effects are assessed as the effects of the manifest group means and individual deviations from the group means (i.e., group mean centering) in multilevel models (Raudenbush and Bryk, 2002). This approach was referred to as the manifest aggregation approach by Marsh et al. (2009) and Lüdtke et al. (2011), which uses manifest aggregation to construct group means. Following the manifest aggregation approach, the MLM (Raudenbush and Bryk, 2002), 2-1-1 mediation, and 1-1-1 mediation (Zhang et al., 2009) models are shown in Table 1.

**TABLE 1:** MLM, 2-1-1 mediation, and 1-1-1 mediation^1^ models in the manifest and latent aggregation approaches.

	Manifest aggregation approach	Latent aggregation approach
MLM	$Y_{ij}=\beta_{00}+\beta_{xw}\left(X_{ij}-{\bar{X}}_{.j}\right)+\beta_{xb}{\bar{X}}_{.j}+u_{0j}+e_{ij}$	*Y*_*ij*_ = *β*_00_ + *β*_*xw*_*X*_*Wij*_ + *β*_*xb*_*X*_*Bj*_ + *u*_0*j*_ + *e*_*ij*_
2-1-1 mediation	$Y_{ij}=\beta_{00}+\beta_{mw}\left(M_{ij}-{\bar{M}}_{.j}\right)+\beta_{mb}{\bar{M}}_{.j}+\beta_{xb}X_{j}+u_{0j}+e_{ij}\left[brback\right]M_{ij}=\alpha_{00}+\alpha_{b}X_{j}+w_{0j}+d_{ij}$	*Y*_*ij*_ = *β*_00_ + *β*_*mw*_*M*_*Wij*_ + *β*_*mb*_*M*_*Bj*_ + *β*_*xb*_*X*_*j*_ + *u*_0*j*_ + *e*_*ij*_[*brback*]*M*_*ij*_ = α_00_ + α_*b*_*X*_*j*_ + *w*_0*j*_ + *d*_*ij*_
1-1-1 mediation	$Y_{ij}=\beta_{00}+\beta_{mw}\left(M_{ij}-{\bar{M}}_{.j}\right)+\beta_{mb}{\bar{M}}_{.j}+\beta_{xw}\left(X_{ij}-{\bar{X}}_{.j}\right)+\beta_{xb}{\bar{X}}_{.j}+u_{0j}+e_{ij}\left[brback\right]M_{ij}=\alpha_{00}+\alpha_{w}\left(X_{ij}-{\bar{X}}_{.j}\right)+\alpha_{b}{\bar{X}}_{.j}+w_{0j}+d_{ij}$	*Y*_*ij*_ = *β*_00_ + *β*_*mw*_*M*_*Wij*_ + *β*_*mb*_*M*_*Bj*_ + *β*_*xw*_*X*_*Wij*_ + *β*_*xb*_*X*_*Bj*_ + *u*_0*j*_ + *e*_*ij*_[*brback*]*M*_*ij*_ = α_00_ + α_*w*_*X*_*Wij*_ + α_*b*_*X*_*Bj*_ + *w*_0*j*_ + *d*_*ij*_

*^1^In the current study, it is assumed that group-level constructs only show group-level effects, and they cannot influence any within-group difference. Y_ij_ is the observed outcome, X_ij_ is the observed predictor, and M_ij_ is the observed mediator of ith individual in the jth group; ${\bar{X}}_{.j}$ is the manifest group mean of X, ${\bar{M}}_{.j}$is the manifest group mean of M, and X_j_ is the observed group-level variable in the jth group; X_Wij_ and X_Bj_ represent the individual-level random component and group-level random component of X for ith individual in jth group, respectively; M_Wij_ and M_Bj_ represent the individual-level random component and group-level random component of M for the ith individual in the jth group respectively; β_00_ is the intercept, β_xw_ and β_xb_ are the within and between effects of X on Y, and β_mw_ and β_mb_ are the within and between effects of M on Y; α_00_ is the intercept, and α_w_ and α_b_ are the within and between effects of X on M; u_0j_ and e_ij_ are group-level and individual-level error terms of Y, and w_0j_ and d_ij_ are group-level and individual-level error terms of M.*

By decomposing *X*_*ij*_ into the uncorrelated group mean ${\bar{X}}_{.j}$ and individual deviation from group mean $\left(X_{ij}-{\bar{X}}_{.j}\right)$, the variance of *X*_*ij*_ seems to be separated into the between-group variance in ${\bar{X}}_{.j}$ and within-group variance in $\left(X_{ij}-{\bar{X}}_{.j}\right)$, and the between and within effects seem to be set apart. However, the manifest group mean ${\bar{X}}_{.j}$ is not a generally perfect or error-free measurement of the group composition^2^. To be specific, with a random sample from group *j*, the sample mean ${\bar{X}}_{.j}$ is not the “true” population mean in the *j*th group, and it involves the sampling error (Lüdtke et al., 2008, 2011). Lüdtke et al. (2008) showed that the expectation of the between-effect estimator of *X* on *Y* in the MLM model was

(1) $$E\left(\beta_{xb}\right)=\frac{\beta_{xb}ICC_{X}+\beta_{xw}\frac{1}{n}\left(1-{\text{ICC}}_{X}\right)}{{\text{ICC}}_{X}+\frac{1}{n}\left(1-{\text{ICC}}_{X}\right)},$$

where β_*xb*_ is the estimator of the between effect of *X* on *Y* in the manifest aggregation approach, ICC_*X*_ is the intraclass correlation coefficient (ICC) of *X*, and *n* is the common group size. Preacher et al. (2010) showed that the expectation of the group-level indirect effect estimator of *X* on *Y* via *M* in the 2-1-1 mediation model was

(2) $$E\left(\alpha_{b}\beta_{mb}\right)=\alpha_{b}\frac{\beta_{mb}\left(\tau_{M}^{2}-\frac{\tau_{XM}^{2}}{\tau_{X}^{2}}\right)+\beta_{mw}\frac{1}{n}\sigma_{M}^{2}}{\left(\tau_{M}^{2}-\frac{\tau_{XM}^{2}}{\tau_{X}^{2}}\right)+\frac{1}{n}\sigma_{M}^{2}},$$

where α_*b*_ and β_*mb*_ are the estimators of the between effects of *X* on *M*, and of *M* on *Y* in the manifest aggregation approach; $\tau_{M}^{2}$ and $\sigma_{M}^{2}$ are the group-level and individual-level variances of *M*; $\tau_{X}^{2}$ is the group-level variance of *X*; and τ_*XM*_ is the group-level covariance between *X* and *M*. Following the logic and assumptions made by Lüdtke et al. (2008) and Preacher et al. (2010), in the 1-1-1 mediation model, the expectation of the group-level indirect effect estimator is

(3) $$\begin{array}{c} E\left(\alpha_{b}\beta_{mb}\right)=\\ \left[\frac{\alpha_{b}\tau_{X}^{2}+\alpha_{w}\frac{1}{n}\sigma_{X}^{2}}{\tau_{X}^{2}+\frac{1}{n}\sigma_{X}^{2}}\right]\\ \left[\frac{\tau_{MY}+\frac{1}{n}\sigma_{MY}-\frac{\left(\tau_{XY}+\frac{1}{n}\sigma_{XY}\right)\left(\tau_{XM}+\frac{1}{n}\sigma_{XM}\right)}{\tau_{X}^{2}+\frac{1}{n}\sigma_{X}^{2}}}{\tau_{M}^{2}+\frac{1}{n}\sigma_{M}^{2}-\frac{{\left(\tau_{XM}+\frac{1}{n}\sigma_{XM}\right)}^{2}}{\tau_{X}^{2}+\frac{1}{n}\sigma_{X}^{2}}}\right], \end{array}$$

where $\sigma_{X}^{2}$ is the individual-level variance of *X*, τ_*MY*_ and σ_*MY*_ are the group-level and individual-level covariances between *M* and *Y*, τ_*XY*_ and σ_*XY*_ are the group-level and individual-level covariances between *X* and *Y*, and σ_*XM*_ is the individual-level covariance between *X* and *M*.

In the three models, unless the within effects are the same as the between effects for all paths (i.e., β_*xw*_ = β_*xb*_, β_*mw*_ = β_*mb*_, and α_*w*_ = α_*b*_), or the individual-level variances and covariances of *X* and *M* are equal to zero, or the group size *n* is infinite, the between-effect estimators are biased. If the within effect is enough to answer the research question, the manifest aggregation approach will provide unbiased estimators. When the between effect is of theoretical interest as it is in the current study, the manifest aggregation approach may not be a good choice. The bias due to sampling error is not only involved in the estimation of the between effect of the decomposed predictor but also affects the estimation of other group-level effects (Lüdtke et al., 2011; Mayer et al., 2014). The sampling error in the aggregated group means and the resulting biased between effect estimators in the manifest aggregation approach are criticized by the latent aggregation approach.

## Latent Aggregation Approach

To correct for the bias in the between-effect estimator due to sampling error, there is a new trend to decompose the between and within effects in a latent aggregation approach (Lüdtke et al., 2008, 2011; Marsh et al., 2009, 2012; Preacher et al., 2010, 2016; Preacher, 2011; Mayer et al., 2014; Ryu, 2015b). This approach models the group-level and individual-level variance–covariance matrices explicitly with multilevel structural equation modeling (MSEM; Muthén, 1990). The group-level and individual-level latent (or random) components are directly modeled to examine the between and within effects. In previous studies, the latent aggregation approach was discussed for the MLM (Lüdtke et al., 2008, 2011), 2-1-1 mediation (Preacher et al., 2010, 2011), and 1-1-1 mediation models (Preacher et al., 2010).

When (1) the model is correctly specified, (2) error terms follow a multivariate normal distribution^3^ with means of zero and constant variances, (3) error terms are uncorrelated with each other as well as the group-level and individual-level latent components of the predictors, (4) group-level latent components are uncorrelated with individual-level latent components, and (5) each group has an infinite population, the latent aggregation approach provides approximately unbiased within and between effects for the MLM (Lüdtke et al., 2008, 2011), 2-1-1 mediation (Preacher et al., 2010, 2011), and 1-1-1 mediation models (Preacher et al., 2010).

The latent aggregation approach outperforms the manifest aggregation approach in the estimation of between effects with sampling error correction under the appropriate assumptions. It has been shown that the between effect estimators in the latent aggregation approach had smaller biases and root mean square error (RMSE) than those in the manifest aggregation approach, when the assumptions in the latent aggregation approach were satisfied (Lüdtke et al., 2008, 2011; Preacher et al., 2011).

However, the latent aggregation approach did not always perform better than the manifest aggregation approach. Intuitively, when the sampling ratio within each group is 100%, the manifest group mean is the population group mean and free from sampling error. The manifest aggregation approach should provide approximately unbiased between effects under this condition. Lüdtke et al. (2008) found that when the within-group sampling ratio approached 100%, the manifest aggregation approach outperformed the latent aggregation approach in the estimation of between effects in the MLM model. Lüdtke et al. (2011) indicated that when only limited information on the group-level construct was available (e.g., a low ICC of predictors, a small number of groups, and a small group size), the manifest aggregation approach could outperform the latent aggregation approach in the RMSE of between-effect estimators in the MLM model. In the 2-1-1 mediation model, McNeish (2017) found that the manifest aggregation approach outperformed the latent aggregation approach with a small number of groups. When the group-level variance components were close to zero, to deal with the unstable between-effect estimators in the latent aggregation approach, Zitzmann et al. (2015) introduced a Bayesian estimation method with a small amount of information in the prior distribution. It was found that the Bayesian estimation provided more accurate between effect estimates in the MLM model than the maximum likelihood (ML) estimation for the latent aggregation approach under the problematic conditions with small group sizes and small ICCs of the predictors. For the doubly latent approach with multiple level-1 indicators, the Bayesian estimation had fewer problems and provided more accurate between-effect estimates than the ML estimation under the conditions with small group sizes and low ICCs (Zitzmann et al., 2016). With challengingly small groups and low ICCs of the predictors, consistent with previous studies, the doubly manifest approach provided more accurate between-effect estimates than the doubly latent approach no matter whether the ML estimation or the Bayesian estimation was used. Under these conditions, the between effect estimates from the doubly latent approach using the Bayesian estimation were between those from the doubly manifest approach and the doubly latent approach using the ML estimation (Zitzmann et al., 2016).

In these previous studies, sometimes the latent aggregation approach performed better in the estimation of between effects, and sometimes the manifest aggregation approach did. The contradiction comes from the different assumptions made by the manifest and latent aggregation approaches: in the manifest aggregation approach, the entire group is assumed to be sampled, or the within-group sampling ratio is assumed to be 100%, while in the latent aggregation approach, the population in each group is assumed to be infinite^4^ or the within-group sampling ratio is assumed to be close to 0. When designing a study, sampling of the entire groups is not generally applied. Sampling with replacement is hardly conducted either, and the number of individuals per group is hardly infinite. When the groups are naturally of small or moderate sizes, like classrooms and schools, not to mention “infinite,” the number of individuals per group is even further away from “large enough.”

This problem was mentioned in some previous studies. Lüdtke et al. (2008) and Preacher et al. (2010) limited their discussion of the latent aggregation approach to the situations where the within-group sampling ratio was low (e.g., lower than 5%). In the cases where the within-group sampling ratio approached 100%, Lüdtke et al. (2008, 2011), Marsh et al. (2009, 2012), and Preacher et al. (2010) suggested that the manifest aggregation approach might be a natural choice. When the within-group sampling ratio was moderate, Lüdtke et al. (2008) and Marsh et al. (2009) suggested that the “best” between-effect estimate was between the estimates in the manifest and latent aggregation approaches.

In addition to the manifest and latent aggregation approach, Shin and Raudenbush (2010) proposed an alternative approach to estimate the contextual effects with the latent “true” cluster means of covariates for an MLM model and a two-level random slope model. As this alternative approach used a similar idea as the latent aggregation approach and assumed an infinite population within each group, it was not discussed further in the current study.

## Within-Group Finite Population Selection and the New Approach

With a probability sample, it is possible to quantify the sampling error with a consideration of the within-group sampling ratio and correct it in the decomposition of between and within effects. When the sampling ratio exceeds 5% of the population, the selection cannot be treated as if it comes from an infinite population (Cochran, 1977), and a correction is needed. The correction made for the finite population selection is called finite population correction (fpc). It is calculated as (1−*n*/*N*), where *n* is the sample size, and *N* is the population size. Intuitively, with a larger sampling ratio, there is more information and less uncertainty about the population mean. The variance of the mean estimator should be smaller than it is with a smaller sampling ratio (Lohr, 2009). For example, with a simple random sampling (SRS) of *n* individuals from a population of *N*, if the population variance of *Y* is *S*^2^, the variance of $\bar{Y}$ in the sample is $\left(1-\frac{n}{N}\right)\frac{S^{2}}{n}$, which is corrected with fpc.

For the clustered data collected following a complex sampling scheme, the traditional hierarchical linear modeling (HLM) or multilevel linear modeling techniques often assumed an infinite population of both level-1 and level-2 units. This assumption might not hold under most conditions. Some research explored the cooperation of fpc with multilevel techniques when level-2 units were of a finite population. For example, Lai et al. (2018) proposed a method to obtain the finite-population-adjusted standard errors of level-1 and level-2 predictors in two-level models. Their simulation results showed that the bias in the unadjusted standard errors was substantial when the level-2 sample size exceeded 10% of the population size, and increased with a larger ICC, a larger number of groups, and a larger average group size. The proposed fpc-adjusted method provided acceptable standard errors when the number of groups was at least 30, and the average group size was at least 10. Svoboda (2020) evaluated an fpc method in two-level models with both continuous and binary predictors. The fpc method generally provided acceptable levels of relative bias in standard error estimates for the continuous predictors. While, for an unbalanced level-2 binary predictor, the fpc-adjusted standard errors were only acceptable when at least 60 groups were sampled.

Different from these studies on the fpc for a finite level-2 population selection in general multilevel modeling, in the decomposition of between and within effects, the within-group population is more likely to be finite, like the population in a school or an organization. As the group-level and individual-level constructs are extracted from individual data, to decompose the between and within effects, a within-group fpc is needed to correct for the sampling errors in the estimation of variances and covariances of the aggregated group constructs. However, neither the manifest nor the latent aggregation approach takes the within-group fpc into sampling error correction. The previous fpc approaches in the general multilevel modeling did not deal with within-group finite population selection issue either.

The MLM, 2-1-1 mediation, and 1-1-1 mediation models can be formulated as MSEM, which are usually estimated by the ML estimation method in the manifest and latent aggregation approaches (Longford and Muthén, 1992; Muthén, 1994, 1997). To incorporate the within-group fpc in the estimation of MSEM for the decomposition of between and within effects in contextual studies, Muthén’s ML-based estimator (MUML) might provide some ideas. As the ML estimation is computationally intensive for MSEM, Muthén (1989;1990) suggested an *ad hoc* estimator, which treated the within and between data in a multiple-group fashion with a fitting function of

(4) $$\begin{array}{c} F=J\{\log\left|\Sigma_{W}+n_{0}\Sigma_{B}\right|+tr({\left[\Sigma_{W}+n_{0}\Sigma_{B}\right]}^{-1}\\ \left[S_{B}+n_{0}(\bar{Z}-\text{μ})(\bar{Z}-\text{μ}{)}^{\prime }])\right\}\\ +(\sum_{j}^{J}n_{j}-J)\{\log\left|\Sigma_{W}\right|+tr(\Sigma_{W}^{-1}S_{PW})\}. \end{array}$$

The common cluster size *n*_0_ is

(5) $$n_{0}=\frac{{\left(\sum_{j}^{J}n_{j}\right)}^{2}-\sum_{j}^{J}n_{j}^{2}}{\left(\sum_{j}^{J}n_{j}\right)\left(J-1\right)},$$

and the pooled within variance–covariance matrix and variance–covariance matrix of group means are

(6) $$S_{PW}=\frac{\sum_{j}^{J}\sum_{i}^{n_{j}}\left(Z_{ij}-{\bar{Z}}_{j}\right){\left(Z_{ij}-{\bar{Z}}_{j}\right)}^{\prime }}{\sum_{j}^{J}\left(n_{j}-1\right)},$$

(7) $$S_{B}=\frac{\sum_{j}^{J}\sum_{i}^{n_{j}}\left({\bar{Z}}_{j}-\bar{Z}\right){\left({\bar{Z}}_{j}-\bar{Z}\right)}^{\prime }}{J-1},$$

where Σ_*W*_ and Σ_*B*_ are the within and between variance–covariance matrices in the model, *n*_*j*_ is the number of individuals in the *j*th group, *J* is the total number of groups in the sample, *Z*_*ij*_ is a *k* × 1 vector of *k* variables used in the model observed from individual *i* in group *j*, ${\bar{Z}}_{j}$ is a *k* × 1 vector of the *j*th group means of *k* variables used in the model, $\bar{Z}$ is a *k* × 1 vector of the grand means of *k* variables used in the model, and μ is a *k* × 1 parameter vector of *k* variables’ means.

This simpler estimator is called MUML (Muthén, 2004), limited information ML estimator, or pseudo-balanced ML estimator (Hox and Maas, 2001). This approach ends up with the same fitting function as ML estimation under a balanced design (Muthén, 1989, 1990). Under an unbalanced design, the common group size is used to “pseudo-balance” the data. The statistical inference of MUML estimators has been derived, and its performance under different sample sizes and ICCs have been examined (Hox and Maas, 2001; Yuan and Hayashi, 2005; Hox et al., 2010; Ryu, 2015a).

With a similar idea as the MUML, some previous research introduced the expected *a posteriori* (EAP)-based estimation, which used ANOVA to get the EAP estimates, and then estimated the between and within effects with the EAP estimates in a stepwise manner (Croon and van Veldhoven, 2007; Zitzmann, 2018; Zitzmann and Helm, 2021). Croon and van Veldhoven (2007) found this stepwise procedure resulted in unbiased estimates of between effects on the group-level outcomes, although it did not maximize the complete likelihood function. In a contextual model, when there was limited information about the level-2 constructs (e.g., a small number of groups and low ICCs), the between effects from the ML estimation tended to be inaccurate. To deal with this problem, Zitzmann (2018) applied a stabilization procedure in the EAP-based estimation, and found that the EAP-based estimation with stabilization was more accurate than the ML estimation for the between effects (Zitzmann, 2018). In addition, Zitzmann and Helm (2021) showed that the EAP-based estimation could also be used for the estimation of complex MSEM with mediation, moderation, and nonlinear effects, and found that the EAP-based estimation had a smaller relative RMSE than the ML estimation, especially when the sample was of small to medium sizes (Zitzmann and Helm, 2021).

Since the MUML estimation showed minor problems in the estimation and could be easily implemented using standard SEM software packages (Ryu, 2015a), it was used for MSEM in empirical studies (Cheung and Au, 2005; Stapleton, 2006; Ryu, 2014, 2015a; Wu et al., 2018). In the MUML, the between and within effects are estimated by treating *S*_*PW*_ and *S*_*B*_ in a multiple-group fashion. It was adopted by Laura M. Stapleton (2002) to incorporate sampling weights into the estimation of MSEM via MUML, in which the weighted *S*_*PW*_ and *S*_*B*_ were used. Following this logic, it is possible to use the within-group fpc in the estimation of *S*_*PW*_ and *S*_*B*_, and estimate the between and within effects via MUML based on the adjusted *S*_*PW*_ and *S*_*B*_ with within-group fpc. To be specific, based on the method of moment (MOM), with a moderate within-group sampling ratio, the adjusted *S*_*PW*_ and *S*_*B*_ from the MOM are $S_{PW\_fpc}=\frac{n-1}{n-fpc}S_{PW}$ and $S_{B\_fpc}=S_{B}+\left(\frac{n-1}{n-fpc}\right)\left(1-fpc\right)S_{PW}$, which can be used in the MUML estimation for the new approach with within-group fpc in the current study.

## The Current Study

The literature review showed that, in the decomposition of between and within effects in contextual models, the manifest and latent aggregation approaches made different assumptions about the within-group sampling in the sampling error correction for the aggregated group constructs. When the entire population was selected within each sampled group, the aggregated group mean was free from sampling error and the manifest aggregation approach was suitable. When the within-group sampling ratio was extremely small (e.g., smaller than 5%), the within-group finite population selection was not a major problem in sampling error correction and the latent aggregation approach was appropriate.

However, when the within-group sampling ratio was moderate, which is commonly seen in contextual studies, the within-group finite population selection was of concern in sampling error correction for the decomposition of between and within effects. The between effect estimators from the manifest aggregation approach may be biased as the sampling error in aggregation is not corrected at all. The between-effect estimators from the latent aggregation approach may also be biased as the sampling error is overcorrected by assuming an infinite group size.

As there was no available approach dealing with the within-group finite population selection in sampling error correction in aggregation, the current study first discussed the within-group fpc in the decomposition of between and within effects. The new approach with within-group fpc using MUML estimation based on the adjusted *S*_*PW*_ and *S*_*B*_ was compared with the manifest and latent aggregation approaches with ML estimation in a Monte Carlo simulation study. An empirical example using the dataset from the Programme for International Student Assessment (PISA) 2012 was also used to illustrate and compare the three analysis approaches.

## Simulation Study

### Methods

A Monte Carlo simulation study was first conducted to compare the performances of the manifest aggregation approach, the latent aggregation approach, and the new approach with within-group fpc in the decomposition of between and within effects for the MLM, 2-1-1 mediation, and 1-1-1 mediation models. To resemble the data structure typically found in contextual studies, an extremely large number of groups with small to moderate group sizes was assumed in the population, and a two-stage cluster sampling design was assumed to be used for data collection in the current simulation study. The conditions manipulated were balanced or unbalanced design (*BAL*), average group size in the population (*N*, 20 and 100), ICC of the predictor *X* or mediator *M* (ICC*_*X*_*/ICC*_*M*_*, 0.05 and 0.25), the ratio of between to within effects of the predictor *X* and/or mediator *M* (*R*_*X*_/*R*_*M*_, 0.10 and 10), number of groups in the sample (*g*, 50 and 200), and within-group sampling ratio (*r*, 0.1, 0.3, 0.5, 0.7, and 0.9).

#### Population

The average group size (*N*) in the population varied at 20 and 100 in the current study. In the balanced case, all groups were of *N* individuals; in the unbalanced case, half of the groups were of $\frac{3}{2}N$ individuals, and the other half of the groups were of $\frac{1}{2}N$ individuals.

##### Population model

The MLM, 2-1-1 mediation, and 1-1-1 mediation models were considered in this simulation, with their ICCs of the predictor *X* or mediator *M*, and ratios of between to within effects manipulated (see Table 1 for the models).

###### Intraclass correlation coefficient

In the MSEM, the ICCs of the decomposed predictors or mediators were of importance (Muthén and Satorra, 1995; Kim et al., 2012; Lachowicz et al., 2015; Hsu et al., 2016), and ranged from 0.05 to 0.50 in previous simulations (Muthén, 1994; Hox and Maas, 2001; Lüdtke et al., 2008, 2011; Hox et al., 2010; Kim et al., 2012; Hsu et al., 2016; Pham, 2018). Considering the ICCs found in the previous simulation and empirical studies, the ICC was set as 0.05 and 0.25 for *X* in the MLM model and the 1-1-1 mediation model, and the ICC was set as 0.05 and 0.25 for *M* in the 2-1-1 mediation model in the current study. The ICC of *Y* was equal to 0.25 across all conditions in the three models, and the ICC of *M* in the 1-1-1 mediation model ranged from 0.20 to 0.25.

###### Ratio of between to within effects

As discussed in the literature review, when the between and within effects were the same, the between effect estimators in the manifest and latent aggregation approaches were approximately unbiased. The research interest in contextual models focused on the between effects which were different from the within effects. In the current simulation study, the within effects were fixed at certain values, with the ratio of between to within effects of *X* (*R*_*X*_) and *M* (*R*_*M*_) being varied at 0.10 and 10.

###### Population model

In the MLM model, μ*_*Y*_* = μ*_*X*_*^5^ = 0, β_00_ = 0, β*_*xw*_* = 0.2, β*_*xb*_* = *R*_*X*_β*_*xw*_*, and *Var*(*X*_*ij*_) = 1. In the 2-1-1 mediation model, μ*_*Y*_* = μ*_*X*_* = μ*_*M*_* = 0, β_00_ = 0, β*_*mw*_* = 0.1, β*_*mb*_* = *R*_*M*_β*_*mw*_*, β*_*xb*_* = 0.2, α*_*b*_* = 0.2, and *Var*(*X*_*j*_) = 1. In the 1-1-1 mediation model, μ*_*Y*_* = μ*_*X*_* = μ*_*M*_* = 0, β_00_ = 0, β*_*mw*_* = 0.05, β*_*mb*_* = *R*_*M*_β*_*mw*_*, β*_*xw*_* = 0.1, β*_*xb*_* = *R*_*X*_β*_*xw*_*, α*_*w*_* = 0.2, α*_*b*_* = *R*_*X*_α*_*w*_*, and *Var*(*X*_*ij*_) = 1. The individual-level and group-level error terms were assumed to follow multivariate normal distributions in the three models. The variances of individual-level and group-level error terms were set at different values for different *R*_*X*_/*R*_*M*_ and ICC*_*X*_*/ICC*_*M*_*. Please see Table 2 for the distributions of the error terms in the three models.

**TABLE 2:** The population distributions of error terms in the multilevel modeling (MLM), 2-1-1 mediation, and 1-1-1 mediation models.

MLM	2-1-1 mediation	1-1-1 mediation
***R*_*X*_/*R*_*M*_ = 0.1 and ICC*_*X*_*/ICC*_*M*_* = 0.05**		
$\left(\begin{array}{c} {\text{υ}}_{0j}\\ {\text{ε}}_{ij} \end{array}\right)\sim MVN\left(\left(\begin{array}{c} 0\\ 0 \end{array}\right),\left(\begin{array}{cc} 1.35 & \\ 0 & 4 \end{array}\right)\right)$	$\left(\begin{array}{c} {\text{ω}}_{0j}\\ {\text{υ}}_{0j}\\ {\text{ε}}_{ij} \end{array}\right)\sim MVN\left(\left(\begin{array}{c} 0\\ 0\\ 0 \end{array}\right),\left(\begin{array}{ccc} 1 & & \\ 0 & 1.36 & \\ 0 & 0 & 4 \end{array}\right)\right)$	$\left(\begin{array}{c} {\text{ω}}_{0j}\\ {\text{υ}}_{0j}\\ {\text{δ}}_{ij}\\ {\text{ε}}_{ij} \end{array}\right)\sim MVN\left(\left(\begin{array}{c} 0\\ 0\\ 0\\ 0 \end{array}\right),\left(\begin{array}{cccc} 1 & & & \\ 0 & 1.34 & & \\ 0 & 0 & 4 & \\ 0 & 0 & 0 & 4 \end{array}\right)\right)$
***R*_*X*_/*R*_*M*_ = 0.1 and ICC*_*X*_*/ICC*_*M*_* = 0.25**		
$\left(\begin{array}{c} {\text{υ}}_{0j}\\ {\text{ε}}_{ij} \end{array}\right)\sim MVN\left(\left(\begin{array}{c} 0\\ 0 \end{array}\right),\left(\begin{array}{cc} 1.34 & \\ 0 & 4 \end{array}\right)\right)$	$\left(\begin{array}{c} {\text{ω}}_{0j}\\ {\text{υ}}_{0j}\\ {\text{ε}}_{ij} \end{array}\right)\sim MVN\left(\left(\begin{array}{c} 0\\ 0\\ 0 \end{array}\right),\left(\begin{array}{ccc} 1 & & \\ 0 & 1.30 & \\ 0 & 0 & 4 \end{array}\right)\right)$	$\left(\begin{array}{c} {\text{ω}}_{0j}\\ {\text{υ}}_{0j}\\ {\text{δ}}_{ij}\\ {\text{ε}}_{ij} \end{array}\right)\sim MVN\left(\left(\begin{array}{c} 0\\ 0\\ 0\\ 0 \end{array}\right),\left(\begin{array}{cccc} 1 & & & \\ 0 & 1.34 & & \\ 0 & 0 & 4 & \\ 0 & 0 & 0 & 4 \end{array}\right)\right)$
***R*_*X*_/*R*_*M*_ = 10 and ICC*_*X*_*/ICC*_*M*_* = 0.05**		
$\left(\begin{array}{c} {\text{υ}}_{0j}\\ {\text{ε}}_{ij} \end{array}\right)\sim MVN\left(\left(\begin{array}{c} 0\\ 0 \end{array}\right),\left(\begin{array}{cc} 1.15 & \\ 0 & 4 \end{array}\right)\right)$	$\left(\begin{array}{c} {\text{ω}}_{0j}\\ {\text{υ}}_{0j}\\ {\text{ε}}_{ij} \end{array}\right)\sim MVN\left(\left(\begin{array}{c} 0\\ 0\\ 0 \end{array}\right),\left(\begin{array}{ccc} 1 & & \\ 0 & 0.24 & \\ 0 & 0 & 4 \end{array}\right)\right)$	$\left(\begin{array}{c} {\text{ω}}_{0j}\\ {\text{υ}}_{0j}\\ {\text{δ}}_{ij}\\ {\text{ε}}_{ij} \end{array}\right)\sim MVN\left(\left(\begin{array}{c} 0\\ 0\\ 0\\ 0 \end{array}\right),\left(\begin{array}{cccc} 1 & & & \\ 0 & 0.89 & & \\ 0 & 0 & 4 & \\ 0 & 0 & 0 & 4 \end{array}\right)\right)$
***R*_*X*_/*R*_*M*_ = 10 and ICC*_*X*_*/ICC*_*M*_* = 0.25**		
$\left(\begin{array}{c} {\text{υ}}_{0j}\\ {\text{ε}}_{ij} \end{array}\right)\sim MVN\left(\left(\begin{array}{c} 0\\ 0 \end{array}\right),\left(\begin{array}{cc} 0.34 & \\ 0 & 4 \end{array}\right)\right)$	$\left(\begin{array}{c} {\text{ω}}_{0j}\\ {\text{υ}}_{0j}\\ {\text{ε}}_{ij} \end{array}\right)\sim MVN\left(\left(\begin{array}{c} 0\\ 0\\ 0 \end{array}\right),\left(\begin{array}{ccc} 1 & & \\ 0 & 0.18 & \\ 0 & 0 & 4 \end{array}\right)\right)$	$\left(\begin{array}{c} {\text{ω}}_{0j}\\ {\text{υ}}_{0j}\\ {\text{δ}}_{ij}\\ {\text{ε}}_{ij} \end{array}\right)\sim MVN\left(\left(\begin{array}{c} 0\\ 0\\ 0\\ 0 \end{array}\right),\left(\begin{array}{cccc} 1 & & & \\ 0 & 0.09 & & \\ 0 & 0 & 4 & \\ 0 & 0 & 0 & 4 \end{array}\right)\right)$

*R_X_/R_M_, between-to-within-effect ratio; ICC_X_/ICC_M_, intraclass correlation coefficient of the decomposed predictor or mediator; υ_0__j_ and ε_ij_, group-level and individual-level population error terms of Y; and ω_0__j_ and δ_ij,_ group-level and individual-level population error terms of M.*

##### Data Generation

In the MLM model, group components $\left(\begin{array}{c} X_{Bj}\\ {\text{υ}}_{0j} \end{array}\right)$ were generated from $\text{MVN}\left(\left(\begin{array}{c} 0\\ 0 \end{array}\right),\left(\begin{array}{cc} {\text{ICC}}_{X} & \\ 0 & Var({\text{υ}}_{0j}) \end{array}\right)\right)$, and *N*_*j*_ individual components $\left(\begin{array}{c} X_{Wij}\\ {\text{ε}}_{ij} \end{array}\right)$ were generated from $\text{MVN}\left(\left(\begin{array}{c} 0\\ 0 \end{array}\right),\left(\begin{array}{cc} 1-{\text{ICC}}_{X} & \\ 0 & Var({\text{ε}}_{ij}) \end{array}\right)\right)$ for each *j*. The mean of *N*_*j*_ *X_*Wij*_* was reset to 0 by using a scale parameter to each *X_*Wij*_* in group *j*, which guaranteed the mean of *X_*Wij*_* in group *j* equal to 0.

In the 2-1-1 mediation model, group components $\left(\begin{array}{c} X_{Bj}\\ {\text{ω}}_{0j}\\ {\text{υ}}_{0j} \end{array}\right)$ were generated from $\text{MVN}\left(\left(\begin{array}{c} 0\\ 0\\ 0 \end{array}\right),\left(\begin{array}{ccc} 1 & & \\ 0 & Var({\text{ω}}_{0j}) & \\ 0 & 0 & Var({\text{υ}}_{0j}) \end{array}\right)\right)$, and *N*_*j*_ individual components $\left(\begin{array}{c} M_{Wij}\\ {\text{ε}}_{ij} \end{array}\right)$ were generated from $\text{MVN}\left(\left(\begin{array}{c} 0\\ 0 \end{array}\right),\left(\begin{array}{cc} \frac{1-{\text{ICC}}_{M}}{{\text{ICC}}_{M}}\times 1.04 & \\ 0 & Var\left(\varepsilon_{ij}\right) \end{array}\right)\right)$ for each *j*. The mean of *N*_*j*_ *M_*Wij*_* was reset to 0 by using a scale parameter to each *M_*Wij*_* in group *j*, which guaranteed the mean of *M_*Wij*_* in group *j* equal to 0.

In the 1-1-1 mediation model, group components $\left(\begin{array}{c} X_{Bj}\\ {\text{ω}}_{0j}\\ {\text{υ}}_{0j} \end{array}\right)$ were generated from $\text{MVN}\left(\left(\begin{array}{c} 0\\ 0\\ 0 \end{array}\right),\left(\begin{array}{ccc} {\text{ICC}}_{X} & & \\ 0 & Var({\text{ω}}_{0j}) & \\ 0 & 0 & Var({\text{υ}}_{0j}) \end{array}\right)\right)$, and *N*_*j*_ individual components $\left(\begin{array}{c} X_{Wij}\\ {\text{δ}}_{ij}\\ {\text{ε}}_{ij} \end{array}\right)$ were generated from $\text{MVN}\left(\left(\begin{array}{c} 0\\ 0\\ 0 \end{array}\right),\left(\begin{array}{ccc} 1-{\text{ICC}}_{X} & & \\ 0 & Var({\text{δ}}_{ij}) & \\ 0 & 0 & Var({\text{ε}}_{ij}) \end{array}\right)\right)$ for each *j*. The mean of *N*_*j*_ *X_*Wij*_* was reset to 0 by using a scale parameter to each *X_*Wij*_* in group *j*, which guaranteed the mean of *X_*Wij*_* in group *j* equal to 0.

#### Sample

##### Number of groups

In the multilevel analysis, a sufficient number of groups was needed for the admissible solutions and asymptotic properties of the between estimators (Kim et al., 2012). In a multilevel factor analysis with MUML, 50 groups were considered as a “small number of groups,” and at least 100 groups were suggested as sufficient for the model test and confidence interval (CI) estimates (Hox and Maas, 2001; Hox et al., 2010). The number of sampled groups (*J*) was set as 50 and 200, and the groups were randomly drawn with equal probability of selection from an infinite population of groups in this study.

##### Within-group sampling ratio

The latent aggregation approach showed an unacceptable bias when the group size was small (e.g., 5), and its efficiency increased with the increase of group size. For a small bias, a group size of 20 was recommended (Preacher et al., 2011). To compare the new approach with within-group fpc to the manifest and latent aggregation approaches, the within-group sampling ratio (*r*) was manipulated from 0.1, 0.3, 0.5, 0.7, to 0.9. For the *j*th group, *n*_*j*_ individuals were randomly drawn from the group of *N*_*j*_, with *n*_*j*_ equal to the product of *N*_*j*_ and within-group sampling ratio *r*.

#### Estimation Method

The simulation was conducted under 2 × 2 × 2 × 2 × 2 × 5 = 160 conditions for each model. Under each condition, the manifest and latent aggregation approaches, as well as the new approach with within-group fpc were applied. The ML was used for the manifest and latent aggregation approaches, and the MUML was used for the new approach. Under each condition, 1,000 replications were conducted.

#### Evaluation Criteria

The parameters of research interests in the current study were the between and within effects of the decomposed predictors and/or mediators. The performances of the three analysis approaches were evaluated in terms of model convergence, accuracy in parameter estimate, variability of the estimator, and accuracy of standard error. The model convergence rate across 1,000 replications was used to evaluate the model convergence for each analysis approach under each simulation condition. The accuracy of the estimator was evaluated by relative bias, which is the average difference between the estimate and population parameter relative to the population parameter over 1,000 replications under each condition. RMSE was used to evaluate the variability of the estimator, which is the square root of the mean square difference between the estimate and parameter over 1,000 replications under each condition. The observed coverage rate reflects the accuracy of standard error in each analysis approach. It is the proportion of times in which the true parameter is in the estimated 95% CI under each condition.

### Results

To evaluate the performances of the manifest aggregation approach, the latent aggregation approach, and the new approach with within-group fpc in the decomposition of between and within effects, the model convergence rate, relative bias, RMSE, and observed coverage rate for the within and between effects were first obtained across the 1,000 replications under each simulation condition for each analysis approach.

As there were 160 simulation conditions for each model using each analysis approach, instead of proving the raw evaluation estimates under each simulation condition, the means and standard deviations of the convergence rate, relative bias, RMSE, and coverage rate across the 160 simulation conditions for each analysis approach were first provided for each parameter. Then, an ANOVA was conducted to examine the contributions of the seven design factors (i.e., analysis approach, *R*_*X*_/*R*_*M*_, ICC*_*X*_*/ICC*_*M*_*, *g*, *BAL*, *N*, and *r*) in explaining the variances of model convergence rate, relative bias, RMSE, and observed coverage rate under different simulation conditions for each parameter. All main and interaction effects were estimated in the ANOVA, and their effect sizes (η^2^) were calculated.

#### Convergence Rate

The three analysis approaches generally showed good model convergence rates for the MLM, 2-1-1 mediation, and 1-1-1 mediation models under most simulation conditions. For the manifest aggregation approach, the convergence rate was close to 100% (*M* = 100.00%, *SD* = 0.01%) for the MLM model, ranged from 96.10 to 100% (*M* = 99.97%, *SD* = 0.31%) for the 2-1-1 mediation model, and from 88.10 to 100% (*M* = 99.69%, *SD* = 1.50%) for the 1-1-1 mediation model across the 160 simulation condition. For the latent aggregation approach, the convergence rate ranged from 91.80 to 100% (*M* = 99.52%, *SD* = 1.32%) for the MLM model, from 85.20 to 100% (*M* = 98.26%, *SD* = 3.44%) for the 2-1-1 mediation model, and from 87.90 to 100% (*M* = 98.59%, *SD* = 2.68%) for the 1-1-1 mediation model. For the new approach with within-group fpc, the convergence rate ranged from 95.20 to 100% (*M* = 99.75%, *SD* = 0.84%) for the MLM model, from 87.50 to 100% (*M* = 99.44%, *SD* = 1.88%) for the 2-1-1 mediation model, and from 84.60 to 100% (*M* = 99.12%, *SD* = 2.77%) for the 1-1-1 mediation model.

The main and interaction effects of the analysis approach did not show any significant or consistent pattern, which largely explained the variance in the convergence rate for the MLM, 2-1-1 mediation, and 1-1-1 mediation models. The non-convergence problems with the latent aggregation approach and the new approach were caused by the non-positive definite estimated between variance-covariance matrices, in which the sampling errors in the aggregation were moved out either without or with within-group fpc. The between variance–covariance matrices in the manifest aggregation approach were estimated using the raw group means, which provided positive definite variance–covariance estimates across all conditions.

#### Bias

As different values were used for different between and within effect parameters in the three models, relative bias was used to evaluate the accuracy in the between and within effect estimates from the three analysis approaches.

##### Within effects

The manifest aggregation approach, the latent aggregation approach, and the new approach with within-group fpc only showed small or negligible differences in the relative biases in within effect estimates in the three models (in Table 3). The main and interaction effects of the analysis approach accounted for less than 5% of the variances in relative biases in these within effect estimates. In other words, there was no significant or consistent pattern of the analysis approach, which largely explained the variance in the relative bias in any within effect estimate.

**TABLE 3:** Relative bias in within and between effect estimates in the MLM, 2-1-1 mediation, and 1-1-1 mediation models.

	MLM		2-1-1 mediation		1-1-1 mediation					
	*M*	*SD*	*M*	*SD*	*M*	*SD*	*M*	*SD*	*M*	*SD*
**Within**	**β*_*xw*_***		**β*_*mw*_***		**β*_*xw*_***		**β*_*mw*_***		**α*_*w*_***	
Manifest	−0.001	0.018	0.000	0.011	−0.002	0.032	−0.005	0.038	−0.002	0.018
Latent	0.010	0.042	0.022	0.056	0.022	0.074	0.008	0.057	0.003	0.023
FPC Latent	0.000	0.019	0.001	0.012	−0.001	0.033	−0.006	0.041	0.000	0.017
**Between**	**β*_*xb*_***		**β*_*mb*_***		**β*_*xb*_***		**β*_*mb*_***		**α*_*b*_***	
Manifest	1.105	2.242	1.175	2.268	1.280	2.528	1.150	1.899	1.098	2.259
Latent	−5.266	16.567	−8.990	31.113	−5.702	20.100	−0.400	2.046	−4.453	14.117
FPC Latent	0.065	1.565	0.291	1.786	0.767	4.483	0.565	1.825	0.165	1.599

*M, the mean of relative bias across the 160 simulation conditions; SD, the standard deviation of relative bias across the 160 simulation conditions; Manifest, manifest aggregation approach; Latent, latent aggregation approach; FPC Latent, the new approach with within-group fpc.*

##### Between effects

As expected, large differences in the between effect estimates were found among the manifest aggregation approach, the latent aggregation approach, and the new approach with within-group fpc (in Table 3). For most between effect estimates in the three models, the new approach showed the smallest degrees of relative biases. On average, the manifest aggregation approach overestimated the between effects, and the latent aggregation approach underestimated the between effects. Different from previous studies on the latent aggregation approach, which assumed the within-group population was infinite (Lüdtke et al., 2008, 2011; Preacher et al., 2011), the current study simulated moderate to large within-group sampling ratios with small to moderate group sizes in the population, which was more in favor of the manifest aggregation approach. For example, when the group size was 20 and within-group sampling ratio was 0.90, the manifest aggregation approach was expected to perform better than the latent aggregation approach in previous studies (Lüdtke et al., 2008, 2011; Marsh et al., 2009, 2012; Preacher et al., 2010). This was reflected in the current results, in which the degrees of relative biases in between effect estimates from the manifest aggregation approach were generally smaller than those from the latent aggregation approach.

**FIGURE 1:**
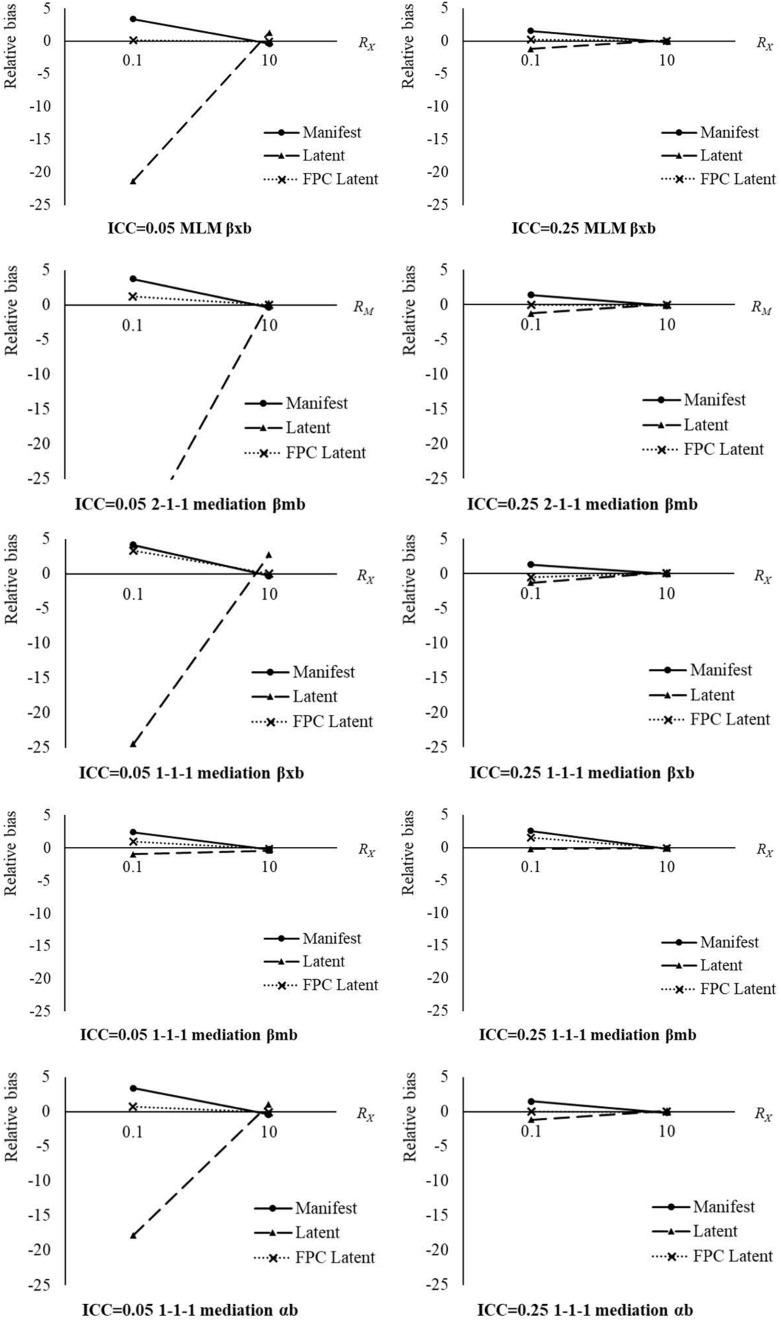
Relative bias in between-effect estimates by analysis approach, between-to-within-effect ratio, and ICC*_*X*_*/ICC*_*M*_*. Manifest, manifest aggregation approach; Latent, latent aggregation approach; FPC Latent, the new approach with within-group fpc; *R*_*X*_/*R*_*M*_, between-to-within-effect ratio; ICC, intraclass correlation coefficient of the decomposed predictor or mediator.

The main effects and interactions of the analysis approach and between-to-within-effect ratio were of medium effect sizes (η^2^s > 0.059) for the relative biases in all between effect estimates in the three models. In addition, the three-way interactions between analysis approach, between-to-within-effect ratio, and ICC*_*X*_*/ICC*_*M*_* were of medium effect sizes (η^2^s > 0.059) in explaining the variation in relative biases of β_*xb*_ in the MLM model, and β_*xb*_ and α*_*b*_* in the 1-1-1 model. The cell means of relative biases in between effect estimates by analysis approach, between-to-within-effect ratio, and ICC*_*X*_*/ICC*_*M*_* are shown in Table 4 and Figure 1. The new approach with within-group fpc generally produced the smallest degrees of relative biases among the three analysis approaches under different between-to-within-effect ratios and ICC*_*X*_*/ICC*_*M*_* for most between effect estimates. In general, the differences in relative biases among the three analysis approaches dropped down with a larger between-to-within-effect ratio and a larger ICC*_*X*_*/ICC*_*M*_*. The degrees of relative biases dropped down when the between-to-within-effect ratio went up from 0.10 to 10, no matter which analysis approach was used. When the between-to-within-effect ratio was 10, the three analysis approaches provided similar relative biases in these between effect estimates. As discussed, the biases in between effect estimates from the manifest and latent aggregation approaches came from the additional parts containing within effects. When the between-to-within-effect ratio was large, i.e., *R*_*X*_/*R*_*M*_ = 10, in the current study, the additional parts containing within effects were relatively small compared to the between effects. Under this condition, the between effect estimates were slightly affected. The degrees of relative biases dropped down when the ICC*_*X*_*/ICC*_*M*_* went up from 0.05 to 0.25, no matter which analysis approach was used. When the ICC*_*X*_*/ICC*_*M*_* was 0.25, the relative biases in these between effect estimates from the three analysis approaches were more similar.

**TABLE 4:** Relative bias in between effect estimates by analysis approach, between-to-within-effect ratio, and ICC*_*X*_*/ICC*_*M*_*.

	MLM		2-1-1 mediation		1-1-1 mediation					
	β*_*xb*_*		β*_*mb*_*		β*_*xb*_*		β*_*mb*_*		α*_*b*_*	
	*R*_*X*_ = 0.1	*R*_*X*_ = 10	*R*_*M*_ = 0.1	*R*_*M*_ = 10	*R*_*X*_ = 0.1	*R*_*X*_ = 10	*R*_*X*_ = 0.1	*R*_*X*_ = 10	*R*_*X*_ = 0.1	*R*_*X*_ = 10
	**ICC*_*X*_*/ICC*_*M*_* = 0.05**									
Manifest	3.392	−0.366	3.768	−0.366	4.152	−0.312	2.370	−0.186	3.407	−0.370
Latent	−21.285	1.333	−35.057	0.225	−24.471	2.762	−0.940	−0.387	−17.813	1.042
FPC Latent	0.117	−0.047	1.173	−0.022	3.360	0.062	0.921	−0.086	0.707	−0.075
	**ICC*_*X*_*/ICC*_*M*_* = 0.25**									
Manifest	1.530	−0.138	1.436	−0.140	1.312	−0.034	2.563	−0.147	1.500	−0.145
Latent	−1.210	0.098	−1.216	0.089	−1.295	0.196	−0.181	−0.092	−1.146	0.105
FPC Latent	0.185	0.003	0.008	0.007	−0.460	0.105	1.521	−0.094	0.031	−0.002

*The mean of relative bias in between effect estimates by between-to-within-effect ratio and ICC of X/M was presented in the table; Manifest, manifest aggregation approach; Latent, latent aggregation approach; FPC Latent, the new approach with within-group fpc; R_X_/R_M_, between-to-within-effect ratio; ICC_X_/ICC_M_, intraclass correlation coefficient of the decomposed predictor or mediator.*

#### Root Mean Square Error

##### Within effects

For the within effect estimates in the three models, the manifest aggregation approach, the latent aggregation approach, and the new approach with within-group fpc provided similar RMSE (in Table 5). From ANOVA, the main effect of the analysis approach and its interaction effects with other design factors explained trivial proportions of variances in RMSE for those within effect estimates. The within-group sampling ratio explained large proportions of variances in RMSE for the within effect estimates in the three models, which ranged from 31 to 44%. The number of groups in the sample, group size, and the interaction between group size and within-group sampling ratio also contributed medium to large proportions to the variances in the RMSE of within effect estimates (η^2^s > 0.059).

**TABLE 5:** Root mean square error of within and between effect estimates in the MLM, 2-1-1 mediation, and 1-1-1 mediation models.

	MLM		2-1-1 mediation		1-1-1 mediation					
	*M*	*SD*	*M*	*SD*	*M*	*SD*	*M*	*SD*	*M*	*SD*
**Within**	**β*_*xw*_***		**β*_*mw*_***		**β*_*xw*_***		**β*_*mw*_***		**α*_*w*_***	
Manifest	0.062	0.053	0.023	0.023	0.063	0.054	0.029	0.024	0.063	0.053
Latent	0.061	0.049	0.022	0.022	0.061	0.050	0.028	0.023	0.061	0.049
FPC Latent	0.066	0.054	0.024	0.024	0.067	0.055	0.031	0.025	0.067	0.054
**Between**	**β*_*xb*_***		**β*_*mb*_***		**β*_*xb*_***		**β*_*mb*_***		**α*_*b*_***	
Manifest	0.439	0.357	0.182	0.186	0.320	0.182	0.102	0.046	0.437	0.361
Latent	1.612	2.071	0.393	0.539	1.962	2.737	0.228	0.206	1.303	1.521
FPC Latent	0.599	0.646	0.178	0.185	0.637	0.659	0.143	0.118	0.533	0.501

*M, the mean of RMSE across the 160 simulation conditions; SD, the standard deviation of RMSE across the 160 simulation conditions; Manifest, manifest aggregation approach; Latent, latent aggregation approach; FPC Latent, the new approach with within-group fpc.*

##### Between effects

For the RMSE in between effect estimates, the three analysis approaches showed large differences. The means and standard deviations of RMSE of between effect estimates across the 160 simulation conditions from the three analysis approaches are presented in Table 5. From the seven-way ANOVA, the analysis approach accounted for medium to large proportions of variances (η^2^s > 0.059) of RMSE in the between effect estimates. For all between effect estimates in the three models, the manifest aggregation approach had the smallest RMSE among the three analysis approaches, while the latent aggregation approach gave the largest ones. The RMSE of between effect estimates from the new approach with within-group fpc were between the statistics from the manifest and latent aggregation approaches.

**FIGURE 2:**
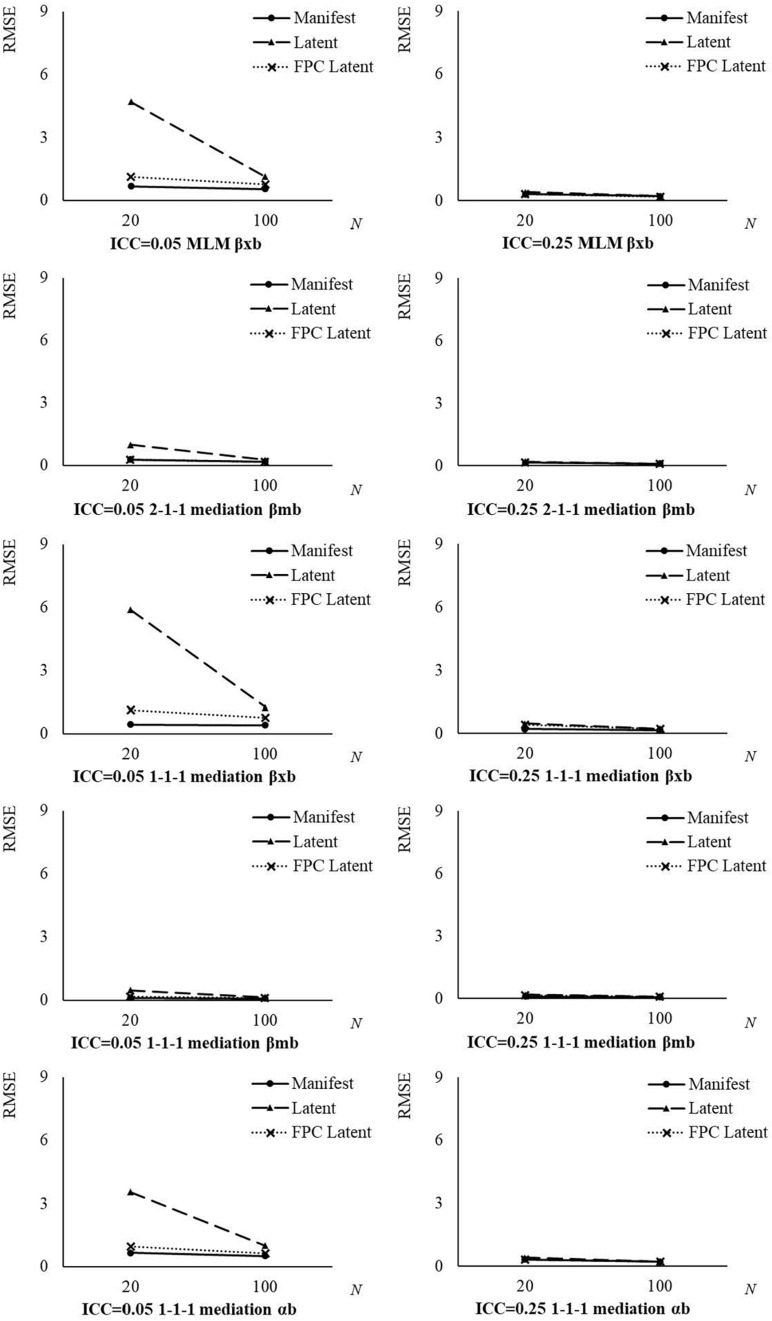
Root mean square error (RMSE) of between effect estimates by analysis approach, ICC*_*X*_*/ICC*_*M*_*, and group size. Manifest, manifest aggregation approach; Latent, latent aggregation approach; FPC Latent, the new approach with within-group fpc; ICC, intraclass correlation coefficient of the decomposed predictor or mediator; *N*, average group size.

From the ANOVA results, the two-way interactions between analysis approach and ICC*_*X*_*/ICC*_*M*_*, and between analysis approach and group size, and the three-way interactions between analysis approach, ICC*_*X*_*/ICC*_*M*_*, and group size, explained medium to large proportions of variances (η^2^s > 0.059) in RMSE of between effect estimates. As shown in Table 6 and Figure 2, the differences in RMSE among the three analysis approaches dropped down with a larger group size and a larger ICC*_*X*_*/ICC*_*M*_*. The RMSE from the latent aggregation approach were more sensitive to the influences of group size and ICC*_*X*_*/ICC*_*M*_*, compared with the other two analysis approaches. No matter which analysis approach was used, the RMSE of between effect estimates were inversely related to the group size and ICC*_*X*_*/ICC*_*M*_*.

**TABLE 6:** Root mean square error of between effect estimates by analysis approach, ICC*_*X*_*/ICC*_*M*_*, and group size.

	MLM		2-1-1 mediation		1-1-1 mediation					
	β*_*xb*_*		β*_*mb*_*		β*_*xb*_*		β*_*mb*_*		α*_*b*_*	
	*N* = 20	*N* = 100	*N* = 20	*N* = 100	*N* = 20	*N* = 100	*N* = 20	*N* = 100	*N* = 20	*N* = 100
	**ICC*_*X*_*/ICC*_*M*_* = 0.05**									
Manifest	0.677	0.547	0.278	0.187	0.456	0.430	0.126	0.099	0.664	0.519
Latent	4.697	1.130	1.001	0.264	5.872	1.253	0.475	0.154	3.562	1.008
FPC Latent	1.125	0.753	0.293	0.168	1.138	0.780	0.189	0.125	0.950	0.651
	**ICC*_*X*_*/ICC*_*M*_* = 0.25**									
Manifest	0.320	0.214	0.160	0.102	0.220	0.174	0.106	0.078	0.336	0.227
Latent	0.409	0.214	0.205	0.101	0.487	0.234	0.184	0.098	0.411	0.230
FPC Latent	0.321	0.196	0.160	0.092	0.415	0.216	0.165	0.093	0.319	0.213

*The mean of RMSE of between effect estimates by average group size and ICC_X_/ICC_M_ was presented in the table; Manifest, manifest aggregation approach; Latent, latent aggregation approach; FPC Latent, the new approach with within-group fpc; ICC_X_/ICC_M_, intraclass correlation coefficient of the decomposed predictor or mediator; N = average group size.*

#### Coverage

As shown in Table 7, the average coverage rates for within effects from the manifest and latent aggregation approaches were close to the nominal level, i.e., 0.95, in the three models. In contrast, the new approach with within-group fpc provided higher coverage rates for the within effects than the nominal level. Its average coverage rates for the within effects were 100%. The differences in coverage rates for within effects among the three analysis approaches were also reflected in the ANOVA results: the largest proportions of variances in coverage rates for within effects were explained by the analysis approach, which were 94, 87, 94, 94, and 67% for the five within effect estimates.

**TABLE 7:** Observed coverage rate for within and between effects in the MLM, 2-1-1 mediation, and 1-1-1 mediation models.

	MLM		2-1-1 mediation		1-1-1 mediation					
	*M*	*SD*	*M*	*SD*	*M*	*SD*	*M*	*SD*	*M*	*SD*
**Within**	**β*_*xw*_***		**β*_*mw*_***		**β*_*xw*_***		**β*_*mw*_***		**α*_*w*_***	
Manifest	0.949	0.008	0.948	0.010	0.949	0.008	0.949	0.008	0.929	0.036
Latent	0.950	0.007	0.947	0.013	0.950	0.007	0.950	0.008	0.949	0.008
FPC Latent	1.000	0.000	1.000	0.000	1.000	0.000	1.000	0.000	1.000	0.000
**Between**	**β*_*xb*_***		**β*_*mb*_***		**β*_*xb*_***		**β*_*mb*_***		**α*_*b*_***	
Manifest	0.767	0.308	0.673	0.371	0.870	0.177	0.842	0.182	0.773	0.302
Latent	0.863	0.178	0.881	0.214	0.924	0.079	0.949	0.047	0.865	0.167
FPC Latent	0.984	0.019	0.987	0.018	0.986	0.019	0.978	0.022	0.984	0.019

*M, the mean of observed coverage rate across the 160 simulation conditions; SD, the standard deviation of observed coverage rate across the 160 simulation conditions; Manifest, manifest aggregation approach; Latent, latent aggregation approach; FPC Latent, the new approach with within-group fpc.*

Different from the results on within effects, the observed coverage rates for the between effects in the new approach with within-group fpc were closer to the nominal level, i.e., 0.95, compared with those from the manifest and latent aggregation approaches (in Table 7). The manifest aggregation approach performed the worst in terms of observed coverage rates for the between effects among the three analysis approaches, with an average observed coverage rate lower than 0.90.

**FIGURE 3:**
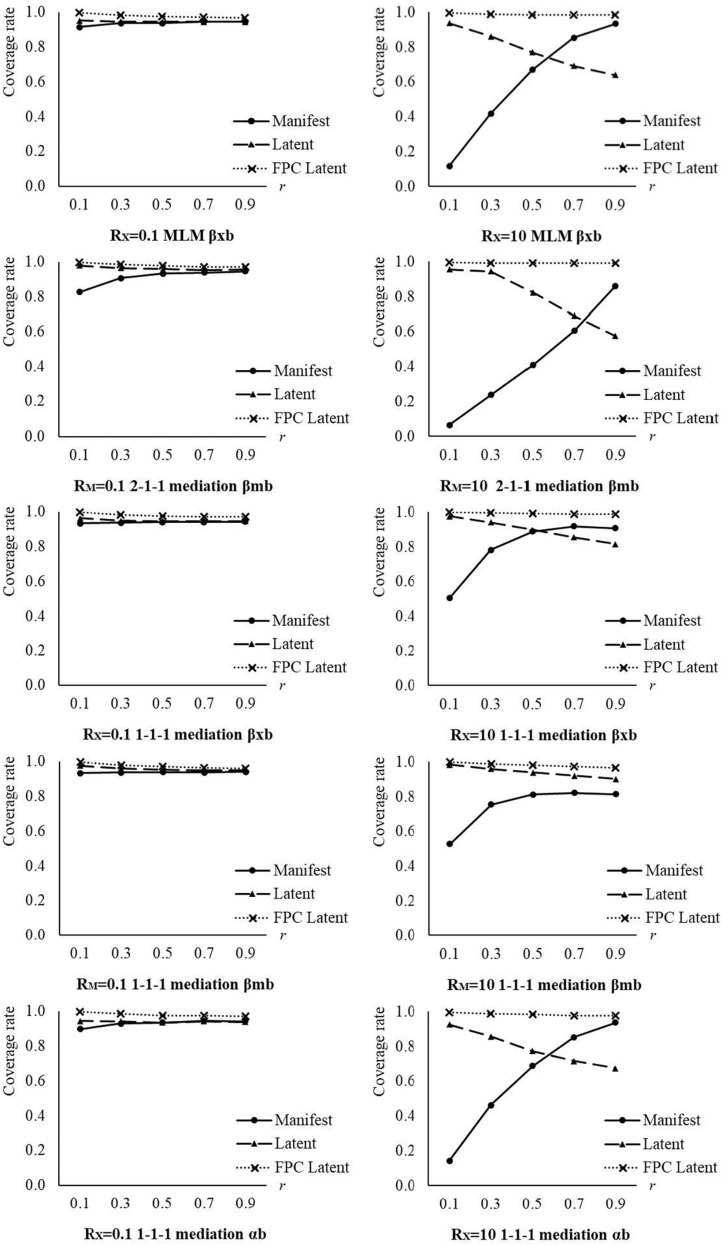
Coverage rate for between effects by analysis approach, *R*_*X*_/*R*_*M*_, and within-group sampling ratio. Manifest, manifest aggregation approach; Latent, latent aggregation approach; FPC Latent, the new approach with within-group fpc; *R*_*X*_/*R*_*M*_, between-to-within-effect ratio; *r*, within-group sampling ratio.

The main effects of analysis approach and between-to-within-effect ratio, the two-way interactions between analysis approach and between-to-within-effect ratio, between analysis approach and within-group sampling ratio, and the three-way interactions between analysis approach, between-to-within-effect ratio, and within-group sampling ratio explained medium to large proportions of variances (η^2^s > 0.059) in coverage rates for these between effects. The cell means of coverage rates for between effects are presented in Table 8 and plotted in Figure 3 by analysis approach, between-to-within-effect ratio, and within-group sampling ratio.

**TABLE 8:** Observed coverage rates for between effects by analysis approach, *R*_*X*_/*R*_*M*_, and within-group sampling ratio.

			MLM	2-1-1 mediation	1-1-1 mediation		
			β*_*xb*_*	β*_*mb*_*	β*_*xb*_*	β*_*mb*_*	α*_*b*_*
		Manifest	0.915	0.827	0.933	0.933	0.899
	*r* = 0.1	Latent	0.952	0.980	0.963	0.976	0.945
		FPC Latent	0.996	0.998	0.997	0.998	0.998
		Manifest	0.938	0.908	0.938	0.938	0.930
	*r* = 0.3	Latent	0.947	0.965	0.949	0.959	0.942
		FPC Latent	0.984	0.987	0.984	0.978	0.986
		Manifest	0.938	0.933	0.941	0.939	0.936
*R*_*X*_/*R*_*M*_ = 0.1	*r* = 0.5	Latent	0.945	0.959	0.946	0.952	0.936
		FPC Latent	0.976	0.978	0.975	0.970	0.977
		Manifest	0.944	0.939	0.941	0.938	0.944
	*r* = 0.7	Latent	0.945	0.954	0.946	0.950	0.943
		FPC Latent	0.973	0.972	0.973	0.964	0.977
		Manifest	0.944	0.946	0.943	0.941	0.942
	*r* = 0.9	Latent	0.944	0.957	0.946	0.951	0.939
		FPC Latent	0.969	0.972	0.970	0.960	0.972
		Manifest	0.116	0.065	0.505	0.528	0.142
	*r* = 0.1	Latent	0.937	0.955	0.976	0.983	0.926
		FPC Latent	0.997	0.994	0.998	1.000	0.997
		Manifest	0.418	0.238	0.783	0.755	0.462
	*r* = 0.3	Latent	0.861	0.944	0.940	0.960	0.857
		FPC Latent	0.990	0.992	0.996	0.989	0.988
		Manifest	0.670	0.408	0.889	0.813	0.688
*R*_*X*_/*R*_*M*_ = 10	*r* = 0.5	Latent	0.770	0.823	0.898	0.939	0.774
		FPC Latent	0.986	0.993	0.991	0.981	0.983
		Manifest	0.853	0.606	0.918	0.822	0.853
	*r* = 0.7	Latent	0.691	0.692	0.856	0.920	0.716
		FPC Latent	0.984	0.993	0.989	0.975	0.979
		Manifest	0.934	0.861	0.907	0.814	0.938
	*r* = 0.9	Latent	0.639	0.576	0.817	0.901	0.675
		FPC Latent	0.983	0.992	0.988	0.967	0.977

*The mean of observed coverage rate for the between effects by between-to-within-effect ratio and within-group sampling ratio was presented in the table; Manifest, manifest aggregation approach; Latent, latent aggregation approach; FPC Latent, the new approach with within-group fpc; R_X_/R_M_, between-to-within-effect ratio; r, within-group sampling ratio.*

The coverage rates for the between effects from the new approach with within-group fpc were above 0.95 in most conditions, and they were much closer to the nominal coverage rate, i.e., 0.95, than those from the manifest and latent aggregation approaches. The coverage rates for the between effects from the manifest and latent aggregation approaches were affected by the between-to-within-effect ratio and within-group sampling ratio. The coverage rates for the between effects from the manifest aggregation approach got better with an increasing within-group sampling ratio, which was consistent with the established findings (Lüdtke et al., 2008, 2011; Marsh et al., 2009, 2012; Preacher et al., 2010). As expected, the coverage rates for the between effects from the latent aggregation approach dropped down with an increasing within-group sampling ratio. The differences in coverage rates for the between effects among the three analysis approaches decreased with a decreasing between-to-within-effect ratio. When the between-to-within-effect ratio was 0.10, the differences in coverage rates among the three analysis approaches (or by different levels of within-group sampling ratio) were trivial. When the between-to-within-effect ratio was 10, the differences in coverage rates among the three analysis approaches (or by different levels of within-group sampling ratio) were clearer. For instance, when the between-to-within-effect ratio was 10 and the within-group sampling ratio was 0.10 or 0.30, the coverage rates for the between effects from the manifest aggregation approach were lower than 80%, which were unacceptable. When the between-to-within-effect ratio was 10 and the within-group sampling ratio was 0.70 and 0.90, the coverage rates for the between effects from the latent aggregation approach were unfavorable. Under the two same conditions, the coverage rates from the new approach were around 95%. The coverage rates for the between effects from the new approach were not affected largely by the between-to-within-effect ratio or within-group sampling ratio, and were better than those from the manifest and latent aggregation approaches.

## Empirical Example

### Background

Since the publication of the Coleman Report (Coleman et al., 1966), continuous efforts have been given to explore the role of schooling in alleviating student SES gap in mathematics performance. Some researchers look at this problem through OTL, which describes students’ content exposures in mathematics and is a key factor to understanding schooling. Previous studies showed OTL had a significant impact on student mathematics achievement, regardless of students’ parental education and income (Cogan et al., 2001; Lleras, 2008; Schmidt et al., 2015). However, the between-school and within-school SES gaps in OTL were found, which exacerbated rather than alleviated SES gaps in student mathematics performance (Schmidt et al., 2015). In other words, high SES schools showed more capabilities to provide advanced mathematics courses to their students, which brought benefits to their student performance on average (Schmidt et al., 2015); within schools, high SES students had more opportunities to attend demanding courses (Roscigno, 1998; Milner, 2012; Reeves, 2012; Kalogrides and Loeb, 2013; Burger, 2016), which were further translated into their advantages in mathematics (Schmidt et al., 2015).

The direct and indirect effects of SES were highly likely to occur at both school-level and student-level, which reflected the institutional-level and individual-level mechanisms. It was necessary to decompose the between and within direct and indirect effects of SES on student mathematics performance via OTL. The main purpose of the current empirical illustration was to show the between and within direct and indirect effects of SES on student mathematics performance through OTL in a 1-1-1 mediation model for different countries using the data from PISA 2012 with the three analysis approaches.

### Methods

Programme for International Student Assessment is an international standardized assessment that measures how well-prepared 15-year-old students are for their future lives (OECD, 2014a, b). In each country (i.e., 34 OECD countries and 31 partner countries in PISA 2012), a stratified two-stage sampling design was used, where schools were sampled using probability proportional to size sampling (PPS), and students were sampled with equal probabilities within sampled schools. There were about 150 schools drawn from each country, with around 30 sampled students within each sampled school. Student weights and school weights were created for the sampled students and schools, which reflected how many other students (or schools) they could represent in the population (OECD, 2014a, b). Based on the student weights and school weights, the school size (of 15-year-olds) and within-school sampling ratio can be calculated as

(8) $$size_{j}=\frac{\sum_{i}^{N_{j}}W\_FSTUWT_{ij}}{W\_FSCHWT_{j}},$$

and

(9) $$ratio_{j}=\frac{n_{j}}{size_{j}},$$

where *size*_*j*_ is the school size (of 15-year-olds) of *j*th school; *N*_*j*_ is total number of sampled students in the *j*th school; *W*_*FSTUWT*_*ij*_ is the student weight (i.e., product of the inverse of the school’s probability of selection and the inverse of the student’s probability of selection within that school) for the *i*th student in the *j*th school; *W*_*FSCHWT*_*j*_ is the school weight (i.e., inverse of the school’s probability of selection) for the *j*th school; *ratio*_*j*_ is the within-school sampling ratio for school *j*; and *n*_*j*_ is the actual number of students in the *j*th school used for analyses^6^.

In PISA, the mean mathematics performance across OECD countries is 494 and the standard deviation is 92. OTL is constructed based on the students’ exposures to 13 mathematics topics. The response categories vary from “never heard of it” to “knew it well.” Student socioeconomic background is represented by the economic, social, and cultural status (ESCS) in PISA. ESCS is computed as a weighted score of students’ home possessions, parents’ occupations, and parents’ education levels. This variable has an average score of 0 and a standard deviation of 1 across OECD countries (OECD, 2014a, b).

In the current study, only students with no missing data on ESCS, OTL, and mathematics performance were included, and only the schools with at least two students were used. After excluding two countries (i.e., Albania and Norway) which did not collect ESCS or OTL information, about two thirds of students in each of the 63 countries were used, as the PISA student survey used a random rotated block design and one third of students had missing data on OTL by design. The number of schools ranged from 12 to 1,421 in these 63 countries, the average within-school sample size ranged from 12 to 81, and the within-school sampling ratio ranged from 0.09 to 0.65.

### Results

In this empirical study, the between and within direct and indirect effects of ESCS on student mathematics performance through OTL were estimated in the 1-1-1 mediation model using the manifest aggregation approach, the latent aggregation approach, and the new approach with within-group fpc for each of the 63 countries in PISA 2012. The results from only five countries are shown in Tables 9, 10 (see Supplementary Materials for the entire results).

**TABLE 9:** Within effects from the manifest aggregation approach, the latent aggregation approach, and the new approach with within-group fpc in the 1-1-1 mediation model.

	β*_*xw*_*			β*_*mw*_*			α*_*w*_*		
	Manifest	Latent	FPC Latent	Manifest	Latent	FPC Latent	Manifest	Latent	FPC Latent
Sweden	26.397	26.380	27.152	4.781	5.567	4.038	0.088	0.089	0.076
	(1.998)	(1.999)	(1.964)	(2.890)	(2.880)	(2.825)	(0.012)	(0.013)	(0.013)
United Kingdom	10.290	10.244	10.029	65.387	65.387	65.229	0.191	0.191	0.190
	(1.040)	(1.041)	(1.042)	(1.273)	(1.273)	(1.277)	(0.009)	(0.009)	(0.009)
Japan	2.622	2.615	2.876	51.905	51.891	52.035	0.060	0.060	0.058
	(1.523)	(1.523)	(1.531)	(2.397)	(2.397)	(2.378)	(0.010)	(0.010)	(0.010)
Germany	6.953	7.122	7.510	42.139	41.965	37.954	0.097	0.096	0.098
	(1.621)	(1.619)	(1.649)	(2.436)	(2.432)	(2.429)	(0.013)	(0.013)	(0.014)
United States	13.495	13.551	13.191	61.420	61.404	60.511	0.178	0.178	0.182
	(1.481)	(1.481)	(1.474)	(2.073)	(2.072)	(2.071)	(0.012)	(0.012)	(0.012)

*Manifest, manifest aggregation approach; Latent, latent aggregation approach; FPC Latent, the new approach with within-group fpc; standard errors are in the parentheses.*

**TABLE 10:** Between effects from the manifest aggregation approach, the latent aggregation approach, and the new approach with within-group fpc in the 1-1-1 mediation model.

	β*_*xb*_*			β*_*mb*_*			α*_*b*_*		
	Manifest	Latent	FPC Latent	Manifest	Latent	FPC Latent	Manifest	Latent	FPC Latent
Sweden	60.129	74.188	68.527	13.194	5.803	8.671	0.291	0.220	0.179
	(5.621)	(8.125)	(8.114)	(8.042)	(14.734)	(12.919)	(0.060)	(0.068)	(0.061)
United Kingdom	53.188	73.318	68.907	74.193	62.260	64.901	0.526	0.613	0.599
	(4.529)	(7.755)	(7.359)	(5.421)	(9.268)	(8.690)	(0.029)	(0.037)	(0.037)
Japan	60.999	67.778	66.495	155.400	160.941	160.304	0.556	0.627	0.606
	(9.919)	(16.741)	(15.878)	(13.481)	(22.275)	(21.238)	(0.035)	(0.040)	(0.042)
Germany	47.214	53.303	50.933	101.002	106.692	106.492	0.527	0.677	0.643
	(5.384)	(11.152)	(10.042)	(6.857)	(13.599)	(12.263)	(0.039)	(0.047)	(0.048)
United States	40.522	40.258	39.850	87.540	104.965	100.782	0.275	0.260	0.262
	(4.938)	(6.636)	(6.406)	(10.558)	(17.606)	(16.175)	(0.032)	(0.035)	(0.035)

*Manifest, manifest aggregation approach; Latent, latent aggregation approach; FPC Latent, the new approach with within-group fpc; standard errors are in the parentheses.*

In most countries, ESCS showed significant direct and indirect within effects via OTL on student mathematics achievement, which was consistent with previous studies (Schmidt et al., 2015). Consistent with the results in the simulation study, the within effect estimates and their standard errors from the three analysis approaches did not differ much from each other in the 63 countries, regardless of the degrees of ICCs of ESCS, OTL, and mathematics performance.

On the school level, significant direct and indirect between effects of ESCS on mathematics performance were also found in most countries, where the magnitudes of the between effects were larger than the corresponding within effects in these countries. Obvious differences in between effect estimates and their standard errors were found among the three analysis approaches. As expected, the between effect estimates and their standard errors from the new approach were generally between the statistics from the manifest and latent aggregation approaches. Different from the results in the simulation study, the between effect estimates from the new approach with within-group fpc were generally closer to the ones from latent aggregation approach than those from the manifest aggregation approach. The difference was related to the within-group sampling ratio in the empirical dataset, which ranged from 0.09 to 0.61, with a mean of 0.31 across these countries, but the within-group sampling ratio was set as 0.10, 0.30, 0.50, 0.70, and 0.90 in the simulation. The fpc was calculated as one minus within-group sampling ratio. With smaller within-group sampling ratios, the between variance-covariance matrices after correction from the new approach were closer to the ones used in the latent aggregation approach.

## Discussion

Contextual effects or compositional effects are of interests in psychological research. The effects of organizational SES, percent of students eligible for free or reduced-price lunch (FRPL), percent of female, and percent of minorities on individual outcomes or development have previously been studied. These group-level compositions are often aggregated from individual data in the sample. To examine their effects on individual outcomes controlling for interindividual differences, traditionally, the manifest aggregation approach is used to separate the between and within effects. Recently, there is a new trend to adopt the latent aggregation approach in the decomposition of between and within effects, in which the sampling error in aggregation is corrected. There are statistical assumptions about the constructs to be decomposed, the population of research interests, and the sampling procedures used for data collection made in both manifest and latent aggregation approaches. However, little attention was given to these assumptions when choosing the analysis approach in the applications.

The current study focused on the decomposition of group compositional effects and individual effects based on the individual data in the sample. To resemble the data structure typically found in empirical research, an extremely large number of groups with small to moderate group sizes was assumed in the population, and a two-stage cluster sampling design with equal selection probability at each sampling stage was assumed. A new approach was proposed to deal with the within-group finite population selection problem in the sampling error correction in aggregation. The performances of the manifest aggregation approach, the latent aggregation approach, and the new approach with within-group fpc were compared in terms of the decomposition of between and within effects in the MLM, 2-1-1 mediation, and 1-1-1 mediation models. An empirical illustration was also used to compare the three analysis approaches in a 1-1-1 mediation model with PISA 2012 dataset.

The results from the simulation study and empirical study were generally in line with the expectations. The three analysis approaches showed acceptable model convergence rates for the three models and little differences in relative bias and RMSE of the within effects. The new approach provided higher coverage rates for the within effects compared to those from the manifest and latent aggregation approaches. For the between effects, large differences were found among the three analysis approaches. From the simulation study, the new approach with within-group fpc outperformed the manifest and latent aggregation approaches in terms of the relative biases and observed coverage rates for the between effects. The manifest aggregation approach had a better performance than the other two approaches in the RMSE for the between effects.

For the point estimates of between effects, the new approach with within-group fpc generally performed the best. The manifest aggregation approach underestimated the between effects, while the latent aggregation approach overestimated the between effects. The empirical study also found similar results, i.e., the between effect estimates from the latent aggregation approach were the largest among the three analysis approaches for most countries, those from the manifest aggregation approach were the smallest, while the between effect estimates from the new approach were in the middle.

On average, the between effect estimators from the manifest aggregation approach were the least variable among the three analysis approaches in the current study. Similarly, Lüdtke et al. (2008) found even with the population and sampling design that favored the latent aggregation approach, the estimates from the manifest aggregation approach were less variable, especially under the conditions with small ICCs, a small number of groups, and a small average group size. However, the accuracies of between effect estimates and their standard error estimates from the manifest aggregation approach were not ideal, which were reflected in its unacceptably low observed coverage rates under a low within-group sample size condition. For example, when the between-to-within-effect-ratio was 10 and the group size was 20, the coverage rates for the between effects from the manifest aggregation approach were around 50% with a within-group sampling ratio of 0.10, and all below 80% with a within-group sampling ratio of 0.30.

Based on these results, the new approach using within-group fpc was preferred, as it provided more accurate parameter and standard error estimates for the between effects than the other two approaches, although the between effect estimators were more variable than those of the manifest aggregation approach.

Previous studies (Lüdtke et al., 2008, 2011; Preacher et al., 2011) paid attention to the reflective group-level constructs, and formative group-level constructs under the conditions with large group sizes and extremely small within-group sampling ratios, and found the latent aggregation approach outperformed the manifest aggregation approach in terms of the bias of between effect estimates. As discussed before, the current study applied moderate to large within-group sampling ratios with small to moderate group sizes in the simulation (or for the formative group-level constructs), which better represented the designs in contextual studies. This population assumption and sampling design did not fit the assumptions made by the latent aggregation approach. It is no surprise that the latent aggregation approach did not show any advantage over the new approach with within-group fpc, and even performed worse than the manifest aggregation approach in the decomposition of between and within effects in the current study, although this result seemed to be completely opposed to the conclusions made before (Lüdtke et al., 2008, 2011; Preacher et al., 2011). Lüdtke et al. (2011) also mentioned that when the within-group sampling ratio was 100%, the manifest aggregation approach would perform better than the latent aggregation approach, and the finite sampling correction was needed when a moderate sampling ratio was used in their study. An additional simulation was conducted for the MLM model in the current study. A moderate group size (i.e., *N* = 100) and an extremely small within-group sampling ratio (i.e., *r* = 0.02) were used for data generation, which generally fit the assumptions made by the latent aggregation approach. Similar to the results from previous studies (Lüdtke et al., 2008, 2011; Preacher et al., 2011), the latent aggregation approach outperformed the manifest aggregation approach in term of biases in between effect estimates under this condition, and the new approach provided similar between effect estimates as those from the latent aggregation approach.

The unstable between effect estimates from the latent aggregation approach was not just related to a moderate to high within-group sampling ratio. Even with an infinite within-group population, it was found that the between effect estimates from the latent aggregation approach using ML estimation might be unstable when the group-level variance components were close to zero. To improve the estimation accuracy in the between effects for the latent aggregation approach under the conditions with a small number of groups and low ICCs, the Bayesian estimation and EAP-based estimation were proposed in the previous studies. It was shown that the between effect estimates in the latent aggregation approach using Bayesian estimation were between the results from the manifest aggregation approach using ML estimation and the results from the latent aggregation approach using ML estimation (Zitzmann et al., 2016). Under the challenging conditions with a small number of groups and low ICCs of predictors, the EAP-based estimation worked better for the between effects than the ML estimation in the latent aggregation approach, and it worked similarly as the ML estimation in other conditions (Croon and van Veldhoven, 2007; Zitzmann, 2018; Zitzmann and Helm, 2021).

The EAP-based estimation used a similar idea as the MUML estimation, both of which separated the between and within effects in a stepwise manner. In the current study, based on the MUML’s idea, a stepwise procedure was conducted in the new approach with within-group fpc. In the first step, the between and within variance-covariance estimates were separated using the ANOVA method. In the second step, the variance-covariance estimates were used to estimate the between and within effects. The major difference between the new approach with within-group fpc in the current study, and the EAP-based estimation in previous studies (Croon and van Veldhoven, 2007; Zitzmann, 2018; Zitzmann and Helm, 2021) was whether they dealt with the within-group finite population selection issue. In the current study, the within-group fpc was incorporated in the ANOVA procedure to get the adjusted between and within variance-covariance estimates in the new approach. Considering the fine performance of EAP-based estimation for the latent aggregation approach (Croon and van Veldhoven, 2007; Zitzmann, 2018; Zitzmann and Helm, 2021) and the within-group fpc for the sampling errors, the new approach with within-group fpc was expected to work better for the between effects than the latent aggregation approach using the ML estimation, especially when there was limited information on the group-level constructs. In the current results, the between effect estimates from the new approach with within-group fpc showed smaller relative biases and smaller RMSE than those from the latent aggregation approach using ML estimation. The differences in between effect estimates between the new approach and the latent aggregation approach were larger when the ICCs of the predictor and/or mediator were smaller and the group size was smaller. The current results did not mean the latent aggregation approach was unfavorable in general but suggested that under the same or a similar population and sampling scenario, the latent aggregation approach might not be a good choice to separate the individual effects and group compositional effects. Furthermore, it would be interesting to compare the between effect estimates from the Bayesian estimation and the EAP-based estimation in the latent aggregation approach proposed before, and those from the new approach with within-group fpc in the current study under different populations and sampling conditions in future studies.

In summary, the results from both simulation and empirical illustration reflect the necessity to consider assumptions about the population and sampling design, as well as the nature of group-level constructs in the decomposition of between and within effects in contextual models. When the within-group sampling ratio is extremely small (e.g., smaller than 5%) or the within-group population can be assumed to be infinite, the latent aggregation approach is a good choice. When the within-group sampling ratio is extremely large (e.g., close to 100%), the manifest aggregation approach can be used to separate the between and within effects. For the contextual studies in which the within-group sampling ratio is usually moderate, the finite population selection needs to be considered in the sampling error correction. Under this condition, the new approach with within-group fpc provides an additional choice to estimate the group compositional effects, with fewer degrees of bias and higher observed coverage rates of between effect estimates compared with those from the manifest and latent aggregation approaches.

### Limitations and Future Study

In the current study, the between and within effects of the decomposed predictors and/or mediators were examined for the three particular two-level models, i.e., MLM, 2-1-1 mediation, and 1-1-1 mediation models, under certain assumptions about the constructs of research interests, populations of subjects, sampling procedures, and the variables used in these models. The results from the simulation could only be generated under the same or similar conditions.

The current study did not cover the conditions which theoretically favored either the manifest or latent aggregation approach. As discussed before, for the formative group-level constructs, when the entire groups are drawn, the manifest aggregation approach is assumed to perform better than the other two approaches. For the reflective group-level constructs under the conditions in which the within-group population can be assumed to be infinite, or for the formative group-level constructs under the conditions with an extremely small within-group sampling ratio (e.g., smaller than 5%), the latent aggregation approach is assumed to be a good choice based on previous studies (Lüdtke et al., 2008, 2011; Preacher et al., 2011). It deserves further simulations to examine the performance of the new approach under these conditions, and compare it with the manifest and latent aggregation approaches.

Second, all models were correctly specified in the current simulation study. It is unclear whether the three-analysis approaches are sensitive to model misspecifications under different settings, or how they will perform under different types of model misspecifications. For example, it was assumed that no omitted confounder influenced either the mediators or the outcomes in the current study. However, in empirical studies, it would be unrealistic or impossible to include and model all the relevant variables. For example, in our empirical illustration, student characteristics (e.g., gender and motivation, etc.), teacher characteristics (e.g., teacher’s degree and major), and school characteristics (e.g., school type and location) were highly likely related to student-level and school-level OTL and outcome, which would bring confounding effects on both direct and indirect effects on the student level and school level. It would be interesting to develop and conduct a sensitivity analysis for the contextual models in order to understand the potential influences of confounders on the between and within effects in the future study. Furthermore, there is no random slope or cross-level interaction discussed in the current study. For the manifest aggregation approach, the random slopes and cross-level interactions can be included in the models following the traditional multilevel modeling strategy (Raudenbush and Bryk, 2002). Marsh et al. (2009) and Preacher et al. (2010, 2011) showed the possibilities to include random slopes and cross-level interactions for the latent aggregation approach. However, when the within effects of the decomposed variables are of random slopes, or there are cross-level interactions involved, the new approach with within-group fpc in the current study cannot be applied. Further work is needed to incorporate the random slopes and cross-level interactions into the new approach. In addition, when the between and within effects of the decomposed variables, as well as the random slopes and cross-level interactions of the within components are all included in the models, it is necessary to reconsider the meaning of these estimates in applications, and whether these analyses and estimation approaches are reasonable for the research questions.

Third, all variables used in the simulation were generated from multivariate normal distributions. With different distributions of variables used in the model, different considerations may be given and different results may come out. In the future study, it is also necessary to consider how to decompose the between and within effects for the variables following distributions other than the multivariate normal distribution, with a correction for the sampling errors in the aggregation.

Moreover, the within-group sampling ratio is necessary for the application of the new approach with within-group fpc. As shown in the empirical study, many large-scale datasets provide the possibilities of calculating the within-group sampling ratios with the available sampling design information. However, different from the current study which assumed a two-stage cluster sampling design with equal selection probability at each stage, the selection probability is not the same either across groups or across individuals in these large-scale datasets. Sampling weights need to be incorporated into estimation for all three-analysis approaches. Previous studies indicated that multilevel pseudo-maximum likelihood estimation (MPML) provides one way to incorporate the group-level and individual-level sampling weights into multilevel models (Asparouhov, 2004, 2006; Rabe-Hesketh and Skrondal, 2006). This estimation method can be adapted and applied to the manifest and latent aggregation approaches. For the new approach with within-group fpc, further studies can work on the incorporation of sampling weights into estimation, with the adjustment of finite population selection in the sampling error correction in aggregation. The comparisons of the three analysis approaches with different weighting procedures in the decomposition of between and within effects are also of research interest.

Furthermore, for the PISA design, balanced repeated replication (BRR) weights should be used to calculate sampling variances in an empirical study. The raw school weights and student weights were only used to estimate the within-school sampling ratio, and all results in the current empirical example were unweighted. As more works are needed, examining the weighting methods for the manifest aggregation approach, the latent aggregation approach, and the new approach with within-group fpc, it also deserves further explorations on the resampling methods to estimate the sampling variances under the complex sampling designs in the decomposition of between and within effects. As the purpose of the current empirical study was to compare the performances of the manifest aggregation approach, the latent aggregation approach, and the new approach under a finite within-group population condition using real data, unweighted results were reported. Further considerations should be given on both weighting methods and replication weights for the three-analysis approaches.

## Data Availability Statement

Publicly available datasets were analyzed in this study. This data can be found here: http://www.oecd.org/pisa/data/.

## Author Contributions

SG designed the study, conducted the simulation and empirical studies, and took a leading role in writing the manuscript. RH and WS contributed to the simulation study and manuscript writing. All authors contributed to the article and approved the submitted version.

## Conflict of Interest

The authors declare that the research was conducted in the absence of any commercial or financial relationships that could be construed as a potential conflict of interest.

## Footnotes

1 For simplicity, the between-group effect and within-group effect are referred as “between effect” and “within effect” in the current study.

2 The focus in the current study is on the sampling error issue in the aggregation of group compositions, and measurement error is not discussed. *X*_*ij*_ is assumed free from measurement error in this study. This assumption is reasonable for some individual characteristics used in this study, like gender and ethnicity. However, for student SES, this assumption is not generally satisfied. When the measurement error is considered in the model, multiple indicators of *X*_*ij*_ are needed. Please see Lüdtke et al. (2011) for further information.

3 The traditional assumption of multivariate normality is not crucial for the asymptotic results. In fact, MSEM framework is general enough to accommodate variables following non-normal distributions, and some software, like Mplus, had built in estimation methods dealing with non-normal data. We put “multivariate normal distribution assumption” here, as it was assumed in the paper by Preacher et al. (2010, 2011) and Lüdtke et al. (2011) we cited. Preacher et al. (2010, 2011) and Lüdtke et al. (2011) also discussed that the assumption of multivariate normality was not crucial.

4 Lüdtke et al. (2008) discussed the assumptions of finite and infinite population in each group and distinguished the formative and reflective group-level constructs. When the group is the referent in aggregation, group-level construct is a reflective measure, and the assumption of infinite population in each group is reasonable. One example is school climate, in which students within a school rate the climate for the target school. Following the domain sampling theory, students are exchangeable and can be assumed infinite in that school. However, it does not fit the contextual studies in which the group construct is a composition of individual characteristics. When the referent is the individual, and individual characteristics are used for aggregation, the group construct is a formative measure, and the population in each group cannot be assumed infinite. One example is school gender ratio, in which students are not exchangeable in terms of their own gender. When the sampling ratio in a school is 100%, manifest gender ratio in each school is free from sampling error. In the current study, group constructs are group compositions, which are aggregated from the individual characteristics. Individual is the referent and within-group population cannot be assumed infinite.

5 μ*_*Y*_*, μ*_*X*_*, and μ*_*M*_* are the population means of *Y*, *X*, and M, respectively.

6 Based on the sampling design in PISA, the BRR should be used for calculating sampling variances. As the purpose of the current example is to compare the three analysis approaches under a finite within-group population condition, the unweighted results were reported in this empirical example and BRR weights were not used. Further considerations should be given on the replication weights in the future study.
