# Supplementary material for: The Decomposition of Between and Within Effects in Contextual Models
Source: Front Psychol. 2021 Jun 3;12:541803. doi: 10.3389/fpsyg.2021.541803 (PMC8209427; doi:10.3389/fpsyg.2021.541803)
Supplement: Supplementary file 1 [file Table_1.docx]

Supplementary Material

Table 11. Within effects from the manifest aggregation approach, the latent aggregation approach, and the new approach with within-group fpc in the 1-1-1 mediation model

| Country | β_xw_ | | | | | | β_mw_ | | | | | | α_w_ | | | | | |
| --- | --- | --- | --- | --- | --- | --- | --- | --- | --- | --- | --- | --- | --- | --- | --- | --- | --- | --- |
|  | Manifest | | Latent | | FPC Latent | | Manifest | | Latent | | FPC Latent | | Manifest | | Latent | | FPC Latent | |
|  | Est | SE | Est | SE | Est | SE | Est | SE | Est | SE | Est | SE | Est | SE | Est | SE | Est | SE |
| Finland | 23.369 | 1.267 | 23.430 | 1.266 | 24.140 | 1.272 | 61.498 | 1.789 | 61.429 | 1.788 | 61.515 | 1.798 | 0.178 | 0.009 | 0.179 | 0.009 | 0.185 | 0.009 |
| Liechtenstein | 6.077 | 4.813 | 5.934 | 4.829 | 9.251 | 4.750 | 24.188 | 6.950 | 24.321 | 6.955 | 22.426 | 7.036 | 0.111 | 0.049 | 0.109 | 0.050 | 0.075 | 0.050 |
| Switzerland | 18.638 | 1.054 | 18.555 | 1.052 | 18.300 | 1.053 | 47.885 | 1.539 | 47.911 | 1.539 | 44.092 | 1.544 | 0.149 | 0.008 | 0.149 | 0.008 | 0.125 | 0.008 |
| Iceland | 20.642 | 2.418 | 20.333 | 2.409 | 21.415 | 2.289 | 19.112 | 2.981 | 18.582 | 2.971 | 13.665 | 2.823 | 0.112 | 0.017 | 0.114 | 0.017 | 0.134 | 0.018 |
| Sweden | 26.397 | 1.998 | 26.380 | 1.999 | 27.152 | 1.964 | 4.781 | 2.890 | 5.567 | 2.880 | 4.038 | 2.825 | 0.088 | 0.012 | 0.089 | 0.013 | 0.076 | 0.013 |
| Canada | 14.845 | 0.805 | 14.849 | 0.805 | 14.633 | 0.801 | 53.287 | 1.084 | 53.335 | 1.084 | 53.074 | 1.076 | 0.156 | 0.006 | 0.157 | 0.006 | 0.152 | 0.006 |
| Netherlands | 3.830 | 1.401 | 3.821 | 1.401 | 3.662 | 1.397 | 43.752 | 1.737 | 43.758 | 1.737 | 43.394 | 1.743 | 0.096 | 0.015 | 0.096 | 0.016 | 0.096 | 0.016 |
| Perm | 17.349 | 3.047 | 17.057 | 3.040 | 17.067 | 2.908 | 42.971 | 4.838 | 42.875 | 4.826 | 42.518 | 4.508 | 0.086 | 0.018 | 0.087 | 0.019 | 0.088 | 0.019 |
| United Kingdom | 10.290 | 1.040 | 10.244 | 1.041 | 10.029 | 1.042 | 65.387 | 1.273 | 65.387 | 1.273 | 65.229 | 1.277 | 0.191 | 0.009 | 0.191 | 0.009 | 0.190 | 0.009 |
| Estonia | 14.974 | 1.785 | 14.715 | 1.780 | 15.234 | 1.799 | 52.612 | 2.923 | 52.789 | 2.916 | 49.391 | 2.984 | 0.084 | 0.011 | 0.085 | 0.011 | 0.079 | 0.011 |
| Denmark | 22.649 | 1.287 | 22.705 | 1.288 | 22.305 | 1.293 | 42.604 | 1.844 | 42.646 | 1.844 | 41.555 | 1.865 | 0.158 | 0.010 | 0.158 | 0.010 | 0.163 | 0.010 |
| Montenegro | 10.545 | 1.433 | 10.491 | 1.431 | 6.624 | 1.446 | 29.987 | 1.886 | 30.033 | 1.883 | 30.389 | 1.842 | 0.083 | 0.014 | 0.083 | 0.014 | 0.076 | 0.014 |
| Jordan | 8.786 | 0.966 | 8.704 | 0.966 | 8.088 | 0.961 | 26.360 | 1.270 | 26.387 | 1.271 | 26.154 | 1.260 | 0.108 | 0.011 | 0.108 | 0.011 | 0.108 | 0.012 |
| Ireland | 14.579 | 1.615 | 14.557 | 1.616 | 14.374 | 1.619 | 56.316 | 2.156 | 56.486 | 2.156 | 56.226 | 2.166 | 0.183 | 0.013 | 0.183 | 0.013 | 0.186 | 0.013 |
| Korea | 8.453 | 1.915 | 8.424 | 1.914 | 9.437 | 1.928 | 70.506 | 2.771 | 70.443 | 2.769 | 72.375 | 2.783 | 0.125 | 0.012 | 0.124 | 0.012 | 0.127 | 0.012 |
| Slovenia | 1.047 | 1.282 | 0.981 | 1.279 | 0.652 | 1.285 | 15.492 | 1.927 | 15.372 | 1.922 | 12.617 | 1.908 | 0.050 | 0.011 | 0.050 | 0.011 | 0.067 | 0.011 |
| Serbia | 7.883 | 1.517 | 7.875 | 1.516 | 6.979 | 1.507 | 34.634 | 2.402 | 34.720 | 2.400 | 35.094 | 2.361 | 0.082 | 0.011 | 0.081 | 0.012 | 0.080 | 0.012 |
| Singapore | 7.366 | 1.504 | 7.380 | 1.504 | 7.568 | 1.502 | 80.063 | 2.058 | 80.045 | 2.058 | 80.051 | 2.058 | 0.173 | 0.012 | 0.173 | 0.012 | 0.171 | 0.012 |
| Croatia | 8.785 | 1.468 | 8.752 | 1.468 | 8.861 | 1.471 | 41.190 | 2.097 | 41.237 | 2.097 | 41.651 | 2.101 | 0.079 | 0.012 | 0.078 | 0.012 | 0.079 | 0.012 |
| Spain | 13.989 | 0.646 | 13.993 | 0.646 | 13.981 | 0.648 | 59.679 | 0.940 | 59.771 | 0.940 | 59.107 | 0.938 | 0.184 | 0.005 | 0.185 | 0.005 | 0.185 | 0.005 |
| Russian Federation | 22.719 | 1.946 | 22.379 | 1.944 | 22.391 | 1.872 | 40.996 | 3.241 | 40.650 | 3.236 | 40.597 | 3.137 | 0.095 | 0.010 | 0.094 | 0.010 | 0.095 | 0.010 |
| Kazakhstan | 10.860 | 1.395 | 10.751 | 1.394 | 10.374 | 1.380 | 19.213 | 1.719 | 19.197 | 1.718 | 19.376 | 1.737 | 0.117 | 0.013 | 0.117 | 0.013 | 0.112 | 0.013 |
| Italy | 3.967 | 0.538 | 4.020 | 0.537 | 3.658 | 0.549 | 29.495 | 0.878 | 29.502 | 0.878 | 27.337 | 0.870 | 0.061 | 0.004 | 0.060 | 0.004 | 0.063 | 0.005 |
| New Zealand | 18.958 | 2.015 | 18.752 | 2.012 | 18.549 | 2.036 | 70.883 | 2.243 | 71.016 | 2.241 | 68.278 | 2.257 | 0.209 | 0.017 | 0.207 | 0.017 | 0.207 | 0.017 |
| Japan | 2.622 | 1.523 | 2.615 | 1.523 | 2.876 | 1.531 | 51.905 | 2.397 | 51.891 | 2.397 | 52.035 | 2.378 | 0.060 | 0.010 | 0.060 | 0.010 | 0.058 | 0.010 |
| Australia | 12.071 | 1.085 | 12.129 | 1.085 | 11.954 | 1.078 | 68.033 | 1.194 | 68.006 | 1.194 | 67.034 | 1.198 | 0.213 | 0.009 | 0.214 | 0.010 | 0.203 | 0.009 |
| Belgium | 8.653 | 1.123 | 8.655 | 1.123 | 8.183 | 1.108 | 53.545 | 1.455 | 53.593 | 1.455 | 52.715 | 1.446 | 0.176 | 0.010 | 0.176 | 0.010 | 0.175 | 0.010 |
| Chinese Taipei | 14.459 | 1.770 | 14.471 | 1.770 | 14.563 | 1.774 | 60.869 | 2.536 | 60.903 | 2.537 | 60.358 | 2.535 | 0.158 | 0.011 | 0.158 | 0.011 | 0.156 | 0.011 |
| Czech Republic | 8.385 | 1.710 | 8.272 | 1.703 | 7.415 | 1.737 | 48.110 | 2.479 | 48.143 | 2.472 | 46.256 | 2.470 | 0.100 | 0.012 | 0.100 | 0.012 | 0.094 | 0.012 |
| Lithuania | 14.426 | 1.651 | 14.249 | 1.651 | 14.661 | 1.653 | 41.982 | 2.660 | 41.429 | 2.656 | 39.954 | 2.577 | 0.078 | 0.011 | 0.078 | 0.012 | 0.068 | 0.012 |
| Latvia | 16.468 | 1.686 | 16.406 | 1.680 | 14.636 | 1.713 | 51.391 | 2.967 | 51.249 | 2.953 | 53.001 | 2.934 | 0.094 | 0.011 | 0.093 | 0.011 | 0.093 | 0.011 |
| Qatar | 3.559 | 1.112 | 3.561 | 1.111 | 2.526 | 1.115 | 29.865 | 1.229 | 29.836 | 1.228 | 28.278 | 1.262 | 0.117 | 0.011 | 0.118 | 0.011 | 0.115 | 0.011 |
| Germany | 6.953 | 1.621 | 7.122 | 1.619 | 7.510 | 1.649 | 42.139 | 2.436 | 41.965 | 2.432 | 37.954 | 2.429 | 0.097 | 0.013 | 0.096 | 0.013 | 0.098 | 0.014 |
| Israel | 15.314 | 1.808 | 15.280 | 1.807 | 15.346 | 1.812 | 54.928 | 2.369 | 55.122 | 2.367 | 50.762 | 2.339 | 0.159 | 0.013 | 0.158 | 0.014 | 0.158 | 0.014 |
| France | 19.650 | 1.726 | 19.620 | 1.726 | 18.626 | 1.703 | 38.699 | 2.595 | 38.396 | 2.594 | 36.888 | 2.477 | 0.115 | 0.012 | 0.115 | 0.013 | 0.127 | 0.013 |
| Malaysia | 10.332 | 1.280 | 10.338 | 1.280 | 10.589 | 1.279 | 45.144 | 1.927 | 45.182 | 1.927 | 44.830 | 1.925 | 0.115 | 0.011 | 0.115 | 0.011 | 0.114 | 0.011 |
| United States | 13.495 | 1.481 | 13.551 | 1.481 | 13.191 | 1.474 | 61.420 | 2.073 | 61.404 | 2.072 | 60.511 | 2.071 | 0.178 | 0.012 | 0.178 | 0.012 | 0.182 | 0.012 |
| Austria | 9.468 | 1.671 | 9.521 | 1.670 | 8.790 | 1.663 | 33.947 | 2.271 | 33.926 | 2.271 | 32.909 | 2.294 | 0.131 | 0.013 | 0.132 | 0.013 | 0.123 | 0.013 |
| Portugal | 17.731 | 1.191 | 17.751 | 1.191 | 18.171 | 1.204 | 48.762 | 2.052 | 48.663 | 2.053 | 46.969 | 2.049 | 0.115 | 0.009 | 0.115 | 0.010 | 0.114 | 0.010 |

Table 11. (cont’d)

| Country | β_xw_ | | | | | | β_mw_ | | | | | | α_w_ | | | | | |
| --- | --- | --- | --- | --- | --- | --- | --- | --- | --- | --- | --- | --- | --- | --- | --- | --- | --- | --- |
|  | Manifest | | Latent | | FPC Latent | | Manifest | | Latent | | FPC Latent | | Manifest | | Latent | | FPC Latent | |
|  | Est | SE | Est | SE | Est | SE | Est | SE | Est | SE | Est | SE | Est | SE | Est | SE | Est | SE |
| Luxembourg | 13.244 | 1.386 | 13.217 | 1.386 | 11.818 | 1.398 | 30.521 | 2.009 | 30.524 | 2.009 | 30.682 | 1.962 | 0.153 | 0.012 | 0.153 | 0.012 | 0.149 | 0.012 |
| Greece | 17.168 | 1.433 | 17.018 | 1.430 | 15.778 | 1.419 | 23.721 | 2.232 | 23.647 | 2.225 | 22.676 | 2.146 | 0.097 | 0.011 | 0.097 | 0.011 | 0.105 | 0.012 |
| United Arab Emirates | 5.966 | 1.036 | 5.912 | 1.035 | 5.457 | 1.032 | 43.893 | 1.200 | 43.914 | 1.199 | 42.580 | 1.185 | 0.120 | 0.010 | 0.121 | 0.010 | 0.115 | 0.010 |
| Poland | 25.153 | 1.763 | 24.941 | 1.765 | 24.194 | 1.764 | 46.391 | 2.607 | 46.698 | 2.608 | 46.477 | 2.607 | 0.116 | 0.012 | 0.117 | 0.012 | 0.115 | 0.012 |
| Turkey | 5.618 | 1.091 | 5.554 | 1.091 | 6.470 | 1.094 | 23.220 | 2.038 | 23.049 | 2.036 | 22.749 | 2.008 | 0.051 | 0.009 | 0.054 | 0.010 | 0.048 | 0.010 |
| Slovak Republic | 18.601 | 1.672 | 18.664 | 1.670 | 20.977 | 1.655 | 47.271 | 2.723 | 46.787 | 2.719 | 51.536 | 2.705 | 0.091 | 0.011 | 0.092 | 0.011 | 0.098 | 0.011 |
| Tunisia | 5.232 | 1.017 | 5.173 | 1.017 | 4.979 | 1.011 | 6.537 | 1.840 | 6.617 | 1.840 | 6.607 | 1.834 | 0.061 | 0.010 | 0.060 | 0.011 | 0.067 | 0.011 |
| Macao-China | 0.091 | 1.569 | 0.084 | 1.568 | -0.186 | 1.559 | 67.258 | 2.251 | 67.320 | 2.251 | 67.798 | 2.276 | 0.097 | 0.012 | 0.097 | 0.012 | 0.112 | 0.011 |
| Hong Kong-China | -1.629 | 1.586 | -1.711 | 1.586 | -2.095 | 1.599 | 44.148 | 2.064 | 44.180 | 2.064 | 43.203 | 2.071 | 0.111 | 0.014 | 0.111 | 0.014 | 0.115 | 0.014 |
| China-Shanghai | 6.655 | 1.553 | 6.656 | 1.553 | 6.802 | 1.556 | 47.228 | 2.970 | 47.212 | 2.970 | 47.128 | 2.979 | 0.081 | 0.009 | 0.081 | 0.009 | 0.084 | 0.009 |
| Brazil | 5.909 | 0.548 | 5.869 | 0.547 | 6.121 | 0.537 | 23.778 | 0.860 | 23.792 | 0.858 | 21.402 | 0.848 | 0.089 | 0.006 | 0.089 | 0.006 | 0.079 | 0.006 |
| Costa Rica | 8.733 | 0.982 | 8.719 | 0.982 | 8.710 | 0.981 | 21.248 | 1.504 | 21.279 | 1.504 | 20.947 | 1.504 | 0.086 | 0.012 | 0.086 | 0.012 | 0.082 | 0.012 |
| Argentina | 7.643 | 1.044 | 7.581 | 1.044 | 7.303 | 1.035 | 19.483 | 1.583 | 19.551 | 1.583 | 20.865 | 1.573 | 0.084 | 0.011 | 0.084 | 0.011 | 0.090 | 0.011 |
| Hungary | 3.246 | 1.372 | 3.115 | 1.367 | 3.360 | 1.381 | 34.551 | 2.329 | 34.658 | 2.321 | 34.269 | 2.293 | 0.053 | 0.010 | 0.053 | 0.011 | 0.065 | 0.011 |
| Indonesia | 3.126 | 0.963 | 3.120 | 0.963 | 3.479 | 0.984 | 11.693 | 1.629 | 11.714 | 1.628 | 12.282 | 1.644 | 0.054 | 0.010 | 0.054 | 0.010 | 0.057 | 0.010 |
| Romania | 12.738 | 1.367 | 12.583 | 1.363 | 11.355 | 1.301 | 22.240 | 1.874 | 22.203 | 1.872 | 23.674 | 1.814 | 0.091 | 0.013 | 0.092 | 0.013 | 0.082 | 0.013 |
| Uruguay | 11.475 | 1.260 | 11.507 | 1.260 | 10.598 | 1.245 | 35.895 | 1.863 | 35.892 | 1.861 | 35.165 | 1.830 | 0.103 | 0.011 | 0.103 | 0.012 | 0.111 | 0.012 |
| Vietnam | 5.601 | 1.206 | 5.552 | 1.206 | 4.549 | 1.210 | 36.377 | 2.402 | 36.245 | 2.400 | 33.067 | 2.310 | 0.038 | 0.009 | 0.038 | 0.009 | 0.038 | 0.009 |
| Colombia | 6.617 | 0.869 | 6.546 | 0.869 | 6.862 | 0.858 | 32.208 | 1.226 | 32.157 | 1.224 | 31.209 | 1.223 | 0.113 | 0.009 | 0.113 | 0.010 | 0.114 | 0.009 |
| Bulgaria | 8.129 | 1.339 | 7.735 | 1.334 | 7.124 | 1.272 | 28.164 | 1.861 | 27.959 | 1.857 | 25.650 | 1.801 | 0.086 | 0.012 | 0.087 | 0.013 | 0.078 | 0.012 |
| Mexico | 3.698 | 0.416 | 3.652 | 0.416 | 3.856 | 0.424 | 29.152 | 0.687 | 29.042 | 0.686 | 28.395 | 0.679 | 0.050 | 0.004 | 0.050 | 0.004 | 0.050 | 0.004 |
| Peru | 7.304 | 1.104 | 7.186 | 1.103 | 7.558 | 1.111 | 30.755 | 1.579 | 30.795 | 1.576 | 29.521 | 1.547 | 0.120 | 0.011 | 0.120 | 0.012 | 0.123 | 0.012 |
| Thailand | 4.912 | 1.060 | 4.687 | 1.061 | 3.917 | 1.045 | 37.877 | 1.970 | 37.492 | 1.970 | 35.844 | 1.907 | 0.082 | 0.008 | 0.083 | 0.008 | 0.075 | 0.008 |
| Chile | 8.145 | 1.233 | 8.025 | 1.235 | 7.831 | 1.218 | 40.295 | 1.808 | 40.390 | 1.809 | 38.955 | 1.760 | 0.080 | 0.010 | 0.079 | 0.010 | 0.079 | 0.011 |

Note. Manifest = manifest aggregation approach; Latent = latent aggregation approach; FPC Latent = the new approach with within-group fpc; *Est=*within effect estimate; *SE=*standard error estimate for the within effect; the 63 countries were sorted by the ICC of ESCS, and their results were presented in this order, in order to compare the within effects from the three analysis approaches.

Table 12. Between effects from the manifest aggregation approach, the latent aggregation approach, and the new approach with within-group fpc in the 1-1-1 mediation model

| Country | β_xb_ | | | | | | β_mb_ | | | | | | α_b_ | | | | | |
| --- | --- | --- | --- | --- | --- | --- | --- | --- | --- | --- | --- | --- | --- | --- | --- | --- | --- | --- |
|  | Manifest | | Latent | | FPC Latent | | Manifest | | Latent | | FPC Latent | | Manifest | | Latent | | FPC Latent | |
|  | Est | SE | Est | SE | Est | SE | Est | SE | Est | SE | Est | SE | Est | SE | Est | SE | Est | SE |
| Finland | 56.297 | 4.826 | 68.628 | 7.108 | 65.418 | 6.914 | 29.314 | 5.014 | 25.028 | 6.182 | 26.313 | 6.158 | 0.085 | 0.057 | 0.003 | 0.084 | 0.011 | 0.078 |
| Liechtenstein | -4.679 | 41.606 | -312.463 | 1446.695 | -7.124 | 97.834 | 123.303 | 44.390 | 344.393 | 1010.804 | 133.734 | 71.853 | 0.535 | 0.212 | 1.375 | 0.338 | 1.136 | 0.313 |
| Switzerland | 64.155 | 6.114 | 99.450 | 9.862 | 88.714 | 8.581 | 33.133 | 5.605 | 20.344 | 6.883 | 21.060 | 6.200 | 0.438 | 0.047 | 0.630 | 0.072 | 0.567 | 0.069 |
| Iceland | 46.385 | 8.380 | 64.741 | 17.175 | 54.820 | 12.278 | 13.734 | 14.837 | 25.776 | 45.983 | 25.673 | 27.472 | 0.209 | 0.045 | 0.266 | 0.067 | 0.234 | 0.063 |
| Sweden | 60.129 | 5.621 | 74.188 | 8.125 | 68.527 | 8.114 | 13.194 | 8.042 | 5.803 | 14.734 | 8.671 | 12.919 | 0.291 | 0.060 | 0.220 | 0.068 | 0.179 | 0.061 |
| Canada | 36.932 | 3.130 | 44.163 | 4.816 | 41.200 | 4.417 | 71.391 | 4.593 | 76.243 | 7.655 | 74.292 | 6.757 | 0.327 | 0.021 | 0.368 | 0.027 | 0.350 | 0.027 |
| Netherlands | 23.330 | 9.077 | 11.497 | 19.082 | 16.429 | 16.902 | 146.101 | 8.015 | 167.820 | 14.760 | 162.204 | 13.247 | 0.728 | 0.065 | 0.987 | 0.086 | 0.935 | 0.088 |
| Perm | 70.378 | 14.830 | 83.882 | 52.976 | 89.118 | 26.779 | 84.779 | 37.463 | 179.470 | 206.123 | 114.576 | 88.185 | 0.203 | 0.044 | 0.239 | 0.056 | 0.219 | 0.060 |
| United Kingdom | 53.188 | 4.529 | 73.318 | 7.755 | 68.907 | 7.359 | 74.193 | 5.421 | 62.260 | 9.268 | 64.901 | 8.690 | 0.526 | 0.029 | 0.613 | 0.037 | 0.599 | 0.037 |
| Estonia | 57.024 | 5.486 | 81.531 | 8.106 | 70.658 | 7.444 | 2.023 | 12.586 | -56.256 | 25.165 | -22.850 | 20.137 | 0.139 | 0.029 | 0.134 | 0.040 | 0.120 | 0.038 |
| Denmark | 54.830 | 3.652 | 69.779 | 6.437 | 60.973 | 5.666 | 36.412 | 6.548 | 18.773 | 13.916 | 30.662 | 11.208 | 0.301 | 0.026 | 0.329 | 0.034 | 0.311 | 0.034 |
| Montenegro | 64.166 | 7.994 | 70.355 | 10.397 | 72.506 | 10.211 | 110.016 | 16.374 | 135.965 | 24.862 | 121.814 | 23.689 | 0.246 | 0.059 | 0.285 | 0.063 | 0.286 | 0.060 |
| Jordan | 35.431 | 4.299 | 43.316 | 5.474 | 43.855 | 5.401 | 66.210 | 6.347 | 75.207 | 8.454 | 70.221 | 8.282 | 0.182 | 0.043 | 0.205 | 0.053 | 0.198 | 0.052 |
| Ireland | 51.369 | 4.551 | 60.371 | 7.453 | 57.532 | 7.106 | 72.202 | 8.269 | 69.638 | 16.209 | 70.618 | 14.849 | 0.335 | 0.033 | 0.358 | 0.039 | 0.340 | 0.041 |
| Korea | 17.795 | 9.109 | -9.115 | 17.592 | -0.130 | 16.029 | 187.427 | 11.856 | 236.253 | 23.101 | 223.059 | 21.013 | 0.574 | 0.042 | 0.654 | 0.046 | 0.636 | 0.047 |
| Slovenia | 77.926 | 6.296 | 110.702 | 16.866 | 94.118 | 12.123 | 92.316 | 9.729 | 83.270 | 27.036 | 94.811 | 19.057 | 0.406 | 0.030 | 0.537 | 0.036 | 0.487 | 0.037 |
| Serbia | 75.762 | 7.219 | 86.863 | 13.424 | 84.198 | 11.891 | 97.194 | 15.306 | 111.814 | 34.480 | 107.208 | 29.514 | 0.271 | 0.032 | 0.312 | 0.036 | 0.299 | 0.036 |
| Singapore | 30.164 | 7.752 | 9.077 | 16.353 | 13.456 | 15.008 | 161.407 | 12.629 | 214.019 | 28.518 | 203.944 | 26.024 | 0.460 | 0.031 | 0.509 | 0.036 | 0.504 | 0.037 |
| Croatia | 51.006 | 6.680 | 43.514 | 11.429 | 46.955 | 10.578 | 144.953 | 12.549 | 190.575 | 24.663 | 177.027 | 22.438 | 0.311 | 0.034 | 0.361 | 0.039 | 0.351 | 0.040 |
| Spain | 30.911 | 2.314 | 35.237 | 3.718 | 32.938 | 3.300 | 66.409 | 4.655 | 63.358 | 8.865 | 64.526 | 7.496 | 0.320 | 0.013 | 0.331 | 0.015 | 0.319 | 0.016 |
| Russian Federation | 56.133 | 7.308 | 71.221 | 19.012 | 69.234 | 10.936 | 46.909 | 19.426 | 66.341 | 82.487 | 61.136 | 38.217 | 0.141 | 0.023 | 0.196 | 0.027 | 0.184 | 0.028 |
| Kazakhstan | 31.208 | 7.589 | 26.775 | 13.902 | 36.089 | 10.683 | 64.631 | 13.676 | 108.510 | 31.899 | 80.174 | 22.022 | 0.280 | 0.033 | 0.333 | 0.039 | 0.320 | 0.039 |
| Italy | 52.449 | 2.783 | 65.489 | 4.130 | 58.259 | 3.807 | 88.979 | 3.799 | 88.079 | 5.157 | 88.215 | 4.734 | 0.395 | 0.017 | 0.511 | 0.022 | 0.474 | 0.022 |
| New Zealand | 60.791 | 6.180 | 87.195 | 11.082 | 77.215 | 10.281 | 67.312 | 7.811 | 42.971 | 14.475 | 54.920 | 13.065 | 0.443 | 0.048 | 0.576 | 0.058 | 0.569 | 0.059 |
| Japan | 60.999 | 9.919 | 67.778 | 16.741 | 66.495 | 15.878 | 155.400 | 13.481 | 160.941 | 22.275 | 160.304 | 21.238 | 0.556 | 0.035 | 0.627 | 0.040 | 0.606 | 0.042 |
| Australia | 42.330 | 3.248 | 49.040 | 5.911 | 47.444 | 5.584 | 91.687 | 3.835 | 96.488 | 7.243 | 95.590 | 6.730 | 0.552 | 0.023 | 0.638 | 0.030 | 0.621 | 0.030 |
| Belgium | 76.403 | 7.041 | 99.199 | 10.593 | 87.936 | 9.680 | 71.283 | 8.065 | 55.595 | 12.015 | 61.990 | 10.715 | 0.610 | 0.039 | 0.677 | 0.044 | 0.641 | 0.046 |
| Chinese Taipei | 64.283 | 8.777 | 55.682 | 14.082 | 57.703 | 13.662 | 177.670 | 14.494 | 213.541 | 24.657 | 207.004 | 23.789 | 0.451 | 0.032 | 0.485 | 0.036 | 0.474 | 0.037 |
| Czech Republic | 73.126 | 6.534 | 110.529 | 12.344 | 90.414 | 10.201 | 99.940 | 8.243 | 77.361 | 14.588 | 91.465 | 12.089 | 0.463 | 0.037 | 0.636 | 0.047 | 0.599 | 0.045 |
| Lithuania | 59.919 | 5.235 | 59.659 | 11.546 | 62.572 | 8.370 | 80.151 | 12.910 | 144.836 | 39.562 | 105.968 | 24.805 | 0.190 | 0.024 | 0.242 | 0.030 | 0.231 | 0.031 |
| Latvia | 51.631 | 4.982 | 69.826 | 10.434 | 60.841 | 7.829 | 54.934 | 12.134 | 32.120 | 31.840 | 49.081 | 21.906 | 0.198 | 0.026 | 0.259 | 0.030 | 0.238 | 0.031 |
| Qatar | 37.188 | 7.221 | 36.247 | 8.111 | 36.470 | 7.342 | 110.649 | 7.958 | 128.032 | 9.393 | 131.178 | 7.830 | 0.499 | 0.064 | 0.507 | 0.069 | 0.406 | 0.074 |
| Germany | 47.214 | 5.384 | 53.303 | 11.152 | 50.933 | 10.042 | 101.002 | 6.857 | 106.692 | 13.599 | 106.492 | 12.263 | 0.527 | 0.039 | 0.677 | 0.047 | 0.643 | 0.048 |
| Israel | 104.104 | 7.818 | 126.564 | 10.009 | 120.648 | 9.922 | 50.129 | 11.841 | 29.167 | 16.520 | 26.745 | 15.736 | 0.307 | 0.045 | 0.339 | 0.052 | 0.312 | 0.052 |
| France | 57.725 | 7.030 | 52.163 | 13.800 | 54.643 | 12.093 | 126.503 | 9.269 | 151.510 | 18.113 | 142.478 | 16.036 | 0.551 | 0.035 | 0.649 | 0.040 | 0.611 | 0.042 |
| Malaysia | 30.509 | 4.671 | 21.887 | 7.383 | 23.616 | 7.135 | 118.274 | 10.811 | 161.027 | 20.711 | 154.183 | 19.694 | 0.267 | 0.027 | 0.284 | 0.030 | 0.281 | 0.030 |
| United States | 40.522 | 4.938 | 40.258 | 6.636 | 39.850 | 6.406 | 87.540 | 10.558 | 104.965 | 17.606 | 100.782 | 16.175 | 0.275 | 0.032 | 0.260 | 0.035 | 0.262 | 0.035 |
| Austria | 30.971 | 9.446 | 31.432 | 14.331 | 30.974 | 12.761 | 89.534 | 9.633 | 94.945 | 13.445 | 91.243 | 12.117 | 0.708 | 0.049 | 0.831 | 0.060 | 0.782 | 0.062 |
| Portugal | 43.121 | 4.225 | 37.906 | 6.493 | 38.845 | 5.613 | 98.447 | 11.524 | 140.660 | 23.042 | 121.186 | 18.770 | 0.199 | 0.023 | 0.213 | 0.024 | 0.207 | 0.025 |

Table 12. (cont’d)

| Country | β_xb_ | | | | | | β_mb_ | | | | | | α_b_ | | | | | |
| --- | --- | --- | --- | --- | --- | --- | --- | --- | --- | --- | --- | --- | --- | --- | --- | --- | --- | --- |
|  | Manifest | | Latent | | FPC Latent | | Manifest | | Latent | | FPC Latent | | Manifest | | Latent | | FPC Latent | |
|  | Est | SE | Est | SE | Est | SE | Est | SE | Est | SE | Est | SE | Est | SE | Est | SE | Est | SE |
| Luxembourg | 65.329 | 10.797 | 75.126 | 14.928 | 73.551 | 11.960 | 33.826 | 21.144 | 19.573 | 30.779 | 27.603 | 25.608 | 0.444 | 0.042 | 0.444 | 0.042 | 0.407 | 0.042 |
| Greece | 48.731 | 5.067 | 35.497 | 11.584 | 47.068 | 8.048 | 89.583 | 12.943 | 189.308 | 41.271 | 124.920 | 25.798 | 0.221 | 0.024 | 0.246 | 0.027 | 0.230 | 0.028 |
| United Arab Emirates | 53.275 | 4.521 | 61.956 | 5.615 | 59.203 | 5.272 | 82.157 | 5.979 | 90.569 | 8.149 | 84.757 | 7.660 | 0.367 | 0.032 | 0.370 | 0.037 | 0.311 | 0.036 |
| Poland | 49.281 | 4.912 | 52.826 | 7.068 | 52.202 | 6.525 | 85.457 | 12.669 | 104.034 | 25.571 | 92.659 | 21.343 | 0.189 | 0.025 | 0.184 | 0.030 | 0.179 | 0.031 |
| Turkey | 48.158 | 7.421 | 40.780 | 11.553 | 48.990 | 10.351 | 104.595 | 16.659 | 182.682 | 33.343 | 165.921 | 27.912 | 0.314 | 0.026 | 0.292 | 0.027 | 0.280 | 0.028 |
| Slovak Republic | 71.581 | 6.685 | 80.681 | 12.528 | 73.765 | 10.312 | 87.618 | 11.209 | 91.043 | 20.777 | 98.939 | 16.637 | 0.425 | 0.028 | 0.506 | 0.032 | 0.502 | 0.033 |
| Tunisia | 39.870 | 4.945 | 41.970 | 7.051 | 44.289 | 6.253 | 65.255 | 18.937 | 104.154 | 44.163 | 81.675 | 35.288 | 0.094 | 0.020 | 0.106 | 0.022 | 0.103 | 0.022 |
| Macao-China | 31.795 | 7.373 | 33.210 | 8.166 | 32.627 | 8.824 | 191.109 | 15.733 | 201.737 | 17.801 | 182.600 | 18.333 | 0.005 | 0.072 | 0.020 | 0.076 | 0.064 | 0.076 |
| Hong Kong-China | 44.979 | 6.495 | 42.261 | 8.578 | 44.920 | 8.275 | 143.936 | 16.758 | 217.395 | 33.520 | 196.147 | 30.455 | 0.148 | 0.030 | 0.151 | 0.032 | 0.151 | 0.033 |
| China-Shanghai | 62.762 | 6.756 | 62.049 | 9.087 | 63.199 | 8.842 | 142.056 | 19.098 | 169.247 | 28.791 | 160.766 | 27.337 | 0.229 | 0.022 | 0.242 | 0.024 | 0.239 | 0.024 |
| Brazil | 19.233 | 2.000 | 13.684 | 3.102 | 16.353 | 2.823 | 83.610 | 3.626 | 105.028 | 5.913 | 96.492 | 5.380 | 0.380 | 0.014 | 0.423 | 0.016 | 0.406 | 0.016 |
| Costa Rica | 24.261 | 3.183 | 21.114 | 5.228 | 22.668 | 4.701 | 59.084 | 7.165 | 77.123 | 13.331 | 69.146 | 11.685 | 0.289 | 0.024 | 0.319 | 0.028 | 0.310 | 0.029 |
| Argentina | 40.330 | 4.119 | 45.845 | 5.704 | 44.285 | 5.208 | 49.165 | 7.694 | 46.268 | 11.167 | 44.105 | 10.013 | 0.361 | 0.026 | 0.391 | 0.029 | 0.377 | 0.030 |
| Hungary | 59.764 | 5.716 | 73.098 | 10.575 | 69.204 | 8.885 | 106.984 | 10.952 | 102.494 | 22.070 | 103.659 | 18.581 | 0.397 | 0.026 | 0.431 | 0.027 | 0.391 | 0.028 |
| Indonesia | 16.568 | 4.348 | 11.031 | 5.988 | 15.301 | 5.560 | 88.272 | 11.652 | 123.218 | 18.532 | 107.261 | 16.160 | 0.193 | 0.022 | 0.218 | 0.024 | 0.220 | 0.025 |
| Romania | 46.808 | 5.324 | 54.227 | 7.493 | 54.022 | 7.131 | 58.847 | 10.135 | 62.770 | 14.849 | 60.477 | 13.616 | 0.333 | 0.028 | 0.374 | 0.035 | 0.369 | 0.036 |
| Uruguay | 33.959 | 3.840 | 29.227 | 5.465 | 30.803 | 5.039 | 85.454 | 7.726 | 105.348 | 12.352 | 98.388 | 11.332 | 0.374 | 0.027 | 0.374 | 0.028 | 0.350 | 0.028 |
| Vietnam | 26.957 | 4.551 | 18.181 | 6.038 | 21.403 | 5.699 | 171.811 | 14.184 | 236.760 | 22.622 | 212.913 | 21.375 | 0.183 | 0.021 | 0.194 | 0.022 | 0.179 | 0.022 |
| Colombia | 29.301 | 2.589 | 29.375 | 3.918 | 31.063 | 3.600 | 58.393 | 5.572 | 70.436 | 9.768 | 65.725 | 8.503 | 0.286 | 0.020 | 0.315 | 0.021 | 0.316 | 0.022 |
| Bulgaria | 41.961 | 5.713 | 44.072 | 11.114 | 49.931 | 8.889 | 83.226 | 12.090 | 113.449 | 26.734 | 99.236 | 20.583 | 0.332 | 0.023 | 0.378 | 0.025 | 0.359 | 0.027 |
| Mexico | 19.788 | 1.098 | 17.640 | 1.476 | 19.227 | 1.384 | 71.505 | 2.929 | 95.337 | 4.721 | 85.793 | 4.156 | 0.196 | 0.009 | 0.211 | 0.009 | 0.205 | 0.009 |
| Peru | 31.589 | 3.320 | 27.475 | 6.277 | 31.442 | 5.153 | 70.391 | 7.932 | 93.420 | 17.067 | 80.156 | 13.495 | 0.307 | 0.019 | 0.330 | 0.020 | 0.326 | 0.020 |
| Thailand | 24.121 | 4.649 | 11.559 | 7.801 | 24.842 | 5.984 | 100.609 | 13.984 | 173.369 | 28.330 | 138.000 | 20.628 | 0.240 | 0.016 | 0.248 | 0.014 | 0.248 | 0.014 |
| Chile | 23.943 | 3.192 | 17.372 | 5.216 | 21.947 | 4.582 | 101.163 | 9.291 | 125.059 | 16.072 | 107.677 | 13.999 | 0.284 | 0.013 | 0.301 | 0.013 | 0.299 | 0.013 |

Note. Manifest = manifest aggregation approach; Latent = latent aggregation approach; FPC Latent = the new approach with within-group fpc; *Est=*between effect estimate; *SE=*standard error estimate for the between effect; the 63 countries were sorted by the ICC of ESCS, and their results were presented in this order, in order to compare the between effects from the three analysis approaches.
